# Genetic Susceptibility to Drug Teratogenicity: A Systematic Literature Review

**DOI:** 10.3389/fgene.2021.645555

**Published:** 2021-04-27

**Authors:** Julia do Amaral Gomes, Emilie Willoch Olstad, Thayne Woycinck Kowalski, Kristina Gervin, Fernanda Sales Luiz Vianna, Lavínia Schüler-Faccini, Hedvig Marie Egeland Nordeng

**Affiliations:** ^1^Programa de Pós-Graduação em Genética e Biologia Molecular (PPGBM), Departamento de Genética, Universidade Federal do Rio Grande do Sul (UFRGS), Porto Alegre, Brazil; ^2^Sistema Nacional de Informação sobre Agentes Teratogênicos (SIAT), Serviço de Genética Médica, Hospital de Clínicas de Porto Alegre (HCPA), Porto Alegre, Brazil; ^3^Laboratório de Medicina Genômica, Centro de Pesquisa Experimental, Hospital de Clínicas de Porto Alegre (HCPA), Porto Alegre, Brazil; ^4^Instituto Nacional de Genética Médica Populacional (INAGEMP), Hospital de Clínicas de Porto Alegre (HCPA), Porto Alegre, Brazil; ^5^Pharmacoepidemiology and Drug Safety Research Group, Department of Pharmacy, Faculty of Mathematics and Natural Sciences, University of Oslo, Oslo, Norway; ^6^PharmaTox Strategic Research Initiative, Faculty of Mathematics and Natural Sciences, University of Oslo, Oslo, Norway; ^7^Complexo de Ensino Superior de Cachoeirinha (CESUCA), Cachoeirinha, Brazil; ^8^Division of Clinical Neuroscience, Department of Research and Innovation, Oslo University Hospital, Oslo, Norway; ^9^Department of Child Health and Development, Norwegian Institute of Public Health, Oslo, Norway

**Keywords:** teratogens, prenatal exposures, pregnancy, congenital anomalies, thalidomide, antidepressant, anticonvulsants, genetic predisposition to disease

## Abstract

Since the 1960s, drugs have been known to cause teratogenic effects in humans. Such teratogenicity has been postulated to be influenced by genetics. The aim of this review was to provide an overview of the current knowledge on genetic susceptibility to drug teratogenicity in humans and reflect on future directions within the field of genetic teratology. We focused on 12 drugs and drug classes with evidence of teratogenic action, as well as 29 drugs and drug classes with conflicting evidence of fetal safety in humans. An extensive literature search was performed in the PubMed and EMBASE databases using terms related to the drugs of interest, congenital anomalies and fetal development abnormalities, and genetic variation and susceptibility. A total of 29 studies were included in the final data extraction. The eligible studies were published between 1999 and 2020 in 10 different countries, and comprised 28 candidate gene and 1 whole-exome sequencing studies. The sample sizes ranged from 20 to 9,774 individuals. Several drugs were investigated, including antidepressants (nine studies), thalidomide (seven studies), antiepileptic drugs (five studies), glucocorticoids (four studies), acetaminophen (two studies), and sex hormones (estrogens, one study; 17-alpha hydroxyprogesterone caproate, one study). The main neonatal phenotypic outcomes included perinatal complications, cardiovascular congenital anomalies, and neurodevelopmental outcomes. The review demonstrated that studies on genetic teratology are generally small, heterogeneous, and exhibit inconsistent results. The most convincing findings were genetic variants in *SLC6A4, MTHFR*, and *NR3C1*, which were associated with drug teratogenicity by antidepressants, antiepileptics, and glucocorticoids, respectively. Notably, this review demonstrated the large knowledge gap regarding genetic susceptibility to drug teratogenicity, emphasizing the need for further efforts in the field. Future studies may be improved by increasing the sample size and applying genome-wide approaches to promote the interpretation of results. Such studies could support the clinical implementation of genetic screening to provide safer drug use in pregnant women in need of drugs.

## Introduction

Congenital anomalies are the leading cause of infant mortality in high-income countries and the second most common cause in many middle-income countries (Sitkin et al., 2015). Such conditions emerge during fetal development and can be inherited or influenced by environmental factors, such as medication exposure (Song and Yosypiv, 2011; Harris et al., 2017; Williams et al., 2019). The prescription of drugs during pregnancy is common, with a prevalence of 40% to 80% in Western countries (Olesen et al., 1999; Engeland et al., 2008; Bakker et al., 2010). Drugs used by the mother during pregnancy can be teratogenic, causing abnormal development of the embryo or fetus. Although such exposures may be preventable, many maternal diseases need to be treated. For example, in cases of cancer, epilepsy, and autoimmune diseases, a drug with known teratogenic potential may be the most effective one, leaving patients and prescribers in a dilemma with challenging treatment decisions (Ostensen, 1992; Bromley et al., 2017; Momen et al., 2017).

Teratogens do not produce congenital anomalies in all exposed embryos, as recognized by Wilson when he proposed his principles of teratogenesis (Wilson, 1977). These principles are still pivotal in the study of teratogenicity and interpretation of teratogenic risk. First, the principles state that the susceptibility to a teratogenic agent depends on the genotype of both the embryo and the mother, as well as the timing of the exposure and the developmental stage of the embryo. In addition, the teratogenic agents act in particular ways on cells or tissues to cause pathogenesis, and the final indications of abnormal development are death, malformation, growth restriction, or functional disorders. Finally, access to the embryo by environmental teratogens depends on the agent's nature, and as the dosage of the agent increases, manifestation of deviant development also increases (Wilson, 1977).

Although little was known about gene-environment interactions and developmental processes at the time Wilson formulated his principles, recent advances in genetics and developmental biology have led to significant improvements in our understanding of the mechanisms of teratogenesis (Yalcin et al., 2015; Griffiths et al., 2018; Piña et al., 2018). Teratogenic events are complex, but an examination of genetic influences on drug response (pharmacogenetics) may promote our understanding of the molecular mechanisms involved (Cassina et al., 2012). This knowledge can be utilized both to identify susceptible individuals by whom the drug should be avoided, and in designing drugs with therapeutic effects and no or minimal teratogenic potential. No recent review of the literature is available in this research field. Therefore, the aim of this systematic review was to provide an overview of the current knowledge on genetic susceptibility to drug teratogenicity in humans and to elucidate future directions within the field.

## Materials and Methods

### Selection of Drugs

Two lists of drugs were created for this review. First, a list of major teratogens was identified based on the book “Drugs during Pregnancy and Lactation: Treatment Options and Risk Assessment” (Schaefer et al., 2014; Appendix 1). This list contains the 12 drugs or classes of drugs that have been recognized across several studies to compromise normal human development *in utero*. A second list was created including 29 drugs and classes of drugs that *may* present a teratogenic or fetotoxic risk to humans according to multiple reviews and original studies (Appendix 2). This list was generated using a wide range of references and discussed among the authors until consensus was reached.

### Eligibility Criteria

The studies included in the review were selected based on the participants, intervention/exposure, comparison group, outcome and study design (PICOS) criteria (Richardson et al., 1995; Eriksen and Frandsen, 2018). Participants were defined as mothers and/or their children exposed to a drug during pregnancy (embryonic/fetal period of development) with known fetal outcome(s). The intervention/exposure was defined as exposure to a drug during pregnancy included in Appendices 1 or 2. The comparison group included mothers and/or their children with different genetic backgrounds. The outcome of interest was the differential occurrence or severity of congenital anomalies or developmental abnormalities based on the association of drug exposure and the genetic background of the individuals. The considered study designs were case-control, cohort, and randomized controlled trials of original articles. Our study was performed in adherence to the Preferred Reporting Items for Systematic Reviews and Meta-Analyses guidelines (PRISMA) (Liberati et al., 2009). Studies in English, Portuguese, and Scandinavian were eligible. No restriction on publication date was set.

Studies were excluded if they had a non-eligible study design (e.g., case reports, studies without a comparison group, studies lacking drug exposure, studies including exposure to many indiscernible drugs, studies assessing drug exposure only after birth, studies with no difference in genetic variation across comparison groups, and *in vitro* or animal model studies), non-eligible outcome (e.g., if congenital anomalies or developmental abnormalities were not evaluated), or if the publication type was non-eligible (e.g., review article, editorial, or conference abstract).

### Search Strategy

Literature searches were performed in the PubMed and EMBASE databases. These databases were selected for the search, as they are the most complete and comprehensive in the fields of medicine and genetics (Khare et al., 2014). The search strategy included: (1) terms associated with the relevant drugs, (2) terms associated with congenital anomalies or developmental abnormalities, and (3) terms associated with genetic variation and genetic susceptibility. The search terms are available in Appendix 3.

Studies published until November 10, 2020, were included in the review. To ensure full coverage of the literature, we performed additional screening of the reference lists for relevant reviews and eligible studies to discover any research articles not identified in the database searches.

### Study Selection

The list of studies generated after searching each database was compiled in EndNote to remove any duplicates. The studies were then imported into Rayyan, a platform for data management in systematic review projects (Ouzzani et al., 2016). The screening and selection of relevant studies was performed independently in two screening rounds by two reviewers (JAG and EWO). In the first round, articles were screened by the titles and abstracts. In the second round, full texts of the articles selected from the first screening were assessed based on the predefined inclusion and exclusion criteria outlined above. Disagreements between reviewers in both phases were resolved by discussion until consensus.

### Data Extraction

The information extracted from each study included the following: (1) study characteristics, including country and study period; (2) design and methodological characteristics, including type of study, sample size, drug of exposure, phenotypic outcomes, and how exposure and outcome were measured; and (3) genetic investigation considerations, including sample tissue, genetic approach, methodology of genetic investigation, genes and genetic variants investigated, and pharmacogenetic outcomes with risk estimates.

## Results

### General Characteristics of the Studies

**Figure 1:**
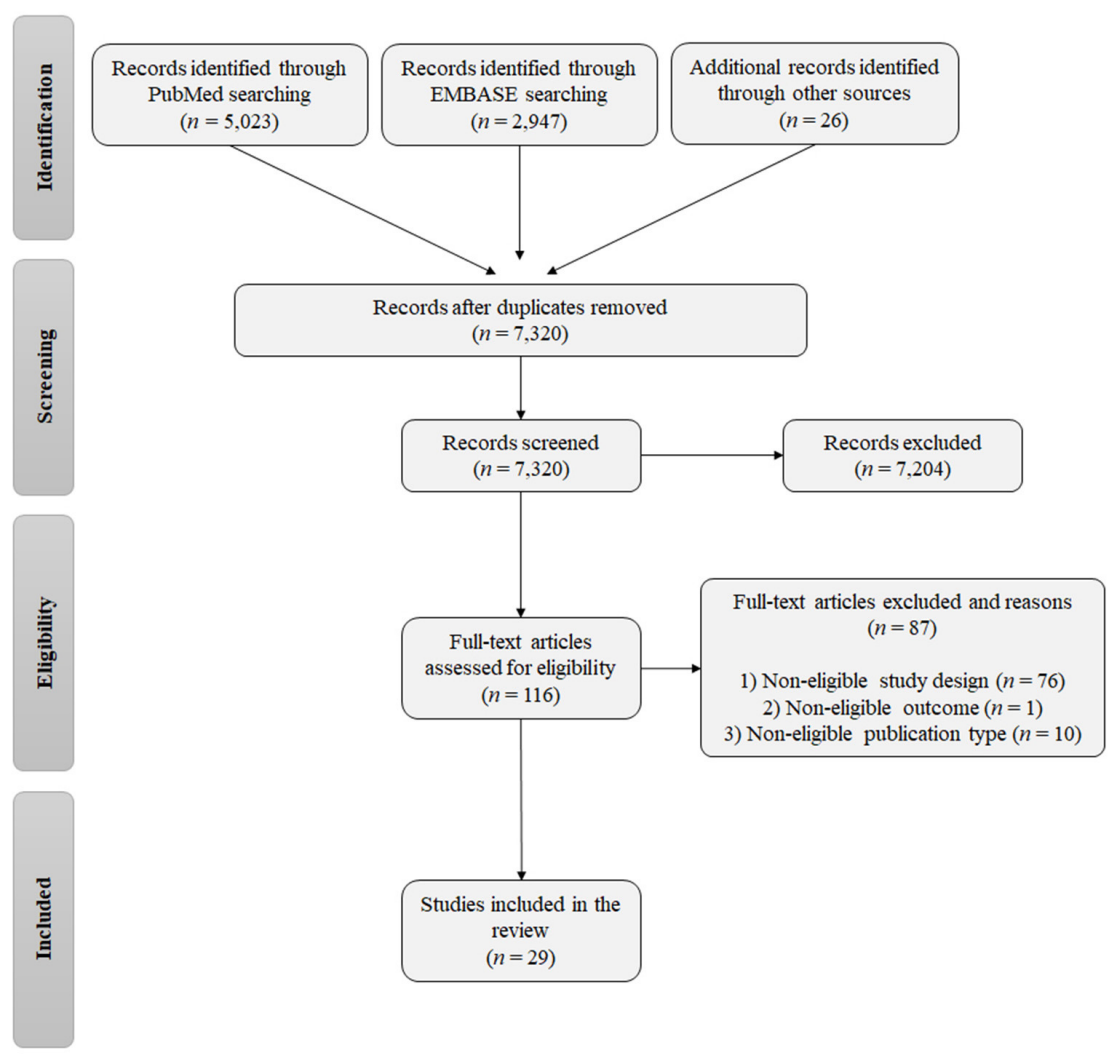
Flowchart of the study screening and selection process.

A total of 7,996 records were identified from the database searches, and the reference lists of relevant reviews and eligible studies (5,023 from PubMed, 2,947 from EMBASE, and 26 from the reference lists of relevant reviews; Figure 1). After removing duplicates, 7,320 studies were eligible for screening of the title and abstract. In the first screening, 7,204 studies were excluded and 116 publications were selected for assessment of the full text. The final list of eligible publications included 29 studies.

The general characteristics of the studies included in this review are presented in Table 1. The studies were published between 1999 and 2020 and were either cohort studies (16 studies) or case-control studies (13 studies). The sample sizes of the studies ranged from 20 to 9,774 individuals broken down as follows: <100 individuals (nine studies), 100–200 individuals (seven studies), 200–800 individuals (nine studies), and >1,000 individuals (four studies). Regarding the individuals, the studies examined neonates (*n* = 8,228), mothers (*n* = 14,481), and fathers (*n* = 105), as well as control individuals (*n* = 935; not exposed to the drugs and unrelated to the cases) and genomic databases (four different databases used as control groups).

**Table 1:** Overview of the reviewed studies.

	**Reference**	**Country; setting (study period)**	**Study design**	**Sample size**	**Drugs of exposure**	**Exposure measures**	**Phenotypic outcomes**	**Outcome measures**
**Antidepressants**	Corti et al. (2019)	Italy (2011–2016)	Case × control	SRIs and maternal depression/anxiety, *n* = 32/30: Normal neonatal genotype, *n* = 10 Altered neonatal genotype, *n* = 22 Normal maternal genotype, *n* = 12 Altered maternal genotype, *n* = 1	Paroxetine, venlafaxine, sertraline, or citalopram	Medical records and follow-up of drug compliance during pregnancy in monthly obstetric and psychiatric visits	Preterm birth, neonatal complications^a^, and umbilical artery pH and base excess	Medical records and follow-up by multidisciplinary team at the hospital
	Ackerman et al. (2017)	USA; sample from the Simons Simplex Collection	Cohort	Antidepressants and LGD mutations *n* = 9 Antidepressants and no LGD mutations *n* = 95 No antidepressants and LGD mutations *n* = 335 No antidepressants and no LGD mutations, *n* = 2,011 (*All participants had ASD*)	Amitriptyline, bupropion, citalopram, escitalopram, fluoxetine, imipramine, paroxetine, sertraline, or venlafaxine	Parent interview; use of antidepressants in ≥1 trimesters	ASD severity	Clinician-measured (ADOS) or parent-reported (ADI-R) ASD severity
	Daud et al. (2017)	The Netherlands; the EUROCAT Northern Netherlands database (1997–2013)	Cohort	SRIs and congenital heart anomaly, *n* = 7 mothers/children No SRIs and congenital heart anomaly, *n* = 28 mothers/children	Fluoxetine, paroxetine, or venlafaxine	Medical records verified by telephone interviews	Congenital heart anomalies	Diagnoses of congenital heart anomalies^b^ before 10 years of age based on ICD-9/10 registered in the EUROCAT Northern Netherlands database
	Nembhard et al. (2017)	USA; the US National Birth Defects Prevention Study (NBDPS; 1997–2008)	Case × control	SSRIs and congenital heart anomaly, *n* = 57 mothers/children SSRIs and no congenital heart anomaly, *n* = 54 mothers/children No SSRIs and congenital heart anomaly, *n* = 1,119 mothers/children No SSRIs and no congenital heart anomaly, *n* = 1,590 mothers/children	Sertraline, fluoxetine, paroxetine, citalopram, or escitalopram	Maternal interview of consecutive drug use for at least 2 months between 2 months before conception and 3 months after conception	Congenital heart anomalies	Diagnoses of congenital heart anomalies based on the classification system specifically developed for the National Birth Defects Prevention Study, incorporating cardiac phenotype, complexity of the phenotype, and extra-cardiac anomalies
	Weikum et al. (2013)	Canada; part of a study on the effects of antenatal SSRI exposure	Cohort	SRIs and maternal mood disorder, *n* = 26 children No SRIs and maternal mood symptoms, *n* = 38 children	Paroxetine, fluoxetine, sertraline, venlafaxine, or citalopram	Physician- or self-reported	Child mood, behavior, and executive functions	Child mood and behavior assessed with the mental health symptomatology section of the MacArthur Health and Behavior Questionnaire. Executive functions assessed by the Hearts and Flowers task
	Brummelte et al. (2013)	Canada (2006–2009)	Cohort	SRIs, *n* = 17 mothers/children No SRIs, *n* = 28/26 mothers/children	Paroxetine, fluoxetine, sertraline, venlafaxine, citalopram, or escitalopram	Medical records on medication, dose, and indication	Levels of reelin in cord blood and maternal blood	Western blot analysis of protein levels in maternal and umbilical cord serum
	Oberlander et al. (2010)	Canada; part of a study on the effects of antenatal SSRI exposure	Cohort	SRIs and maternal mood disorder, *n* = 33 children No SRIs and maternal mood symptoms, *n* = 42 children	Paroxetine, fluoxetine, sertraline, venlafaxine, or citalopram	Physician- or self-reported	Child behavior	Child behavior was evaluated with the Child Behavior Checklist at 3 years of age
	Hilli et al. (2009)	Finland; part of a controlled prospective study by Laine et al., 2003 (1997–2000)	Cohort	SSRIs, *n* = 20 children	Citalopram or fluoxetine	Medical records on the medication, dose, and indication	Perinatal serotonergic symptoms and neurotransmitter blood concentrations	Assessment of serotonergic symptoms by pediatricians (Sternbach, 1991). Neurotransmitter concentrations measured from whole blood and umbilical vein blood samples
	Oberlander et al. (2008)	Canada; part of a study on the effects of antenatal SSRI exposure	Cohort	SRIs, *n* = 37 mothers/children No SRIs, *n* = 47 mothers/children	Paroxetine, fluoxetine, sertraline, venlafaxine, or citalopram	Medical records on medication and indication	Preterm birth and adverse neonatal outcomes (e.g., birth weight, Apgar score, muscle tone, respiratory distress, and jitteriness)	Medical records and follow-up at the hospital
**Antiepileptic drugs**	Jose et al. (2014)	India; part of the Kerala Registry of Epilepsy and Pregnancy (KREP)	Case × control	AEDs, maternal epilepsy and children with congenital anomalies, *n* = 143 mothers AEDs, maternal epilepsy and children without congenital anomalies, *n* = 123 mothers	Carbamazepine, valproic acid, phenytoin, clonazepam, or phenobarbital	Medical records of the registry	Congenital anomalies (main focus on cardio-vascular anomalies)	Clinical examination of malformations at birth. Echocardiography and abdomen ultrasonography at 3 months of age
	Azzato et al. (2010)	USA; part of the Collaborative Perinatal Project (CPP) database (1959–1966)	Cohort	AEDs, *n* = 155/174 (mothers/children)	Phenytoin	Questionnaire data and maternal medical records	Craniofacial anomalies	Craniofacial anomalies considered if microcephaly, abnormal sutures, midfacial hypoplasia, cleft lip, palate or gum, or deformed/low-set ears were noted in medical records. A standardized evaluation form assessing the presence of anomalies was subsequently completed by physicians
	Dean et al. (2007)	Scotland (1976–2002)	Case × control	AEDs, *n* = 276/172/86 (children/mothers/fathers) No AEDs, *n* = 646 healthy blood donors	Valproic acid, phenobarbitone, carbamazepine, phenytoin, lamotrigine, or polytherapy	Medical records and maternal interviews on AED use	Fetal anticonvulsant syndrome, major malformations, and neurodevelopmental disorders	Facial photographs reviewed by a physician for fetal anticonvulsant syndrome (diagnosed if ≥1 major and ≥1 minor feature appeared in the child, or if (s)he had ≥3 minor features). Children without fetal anticonvulsant syndrome were included in analyses of major malformations and neurodevelopmental disorders
	Kini et al. (2007)	United Kingdom (2000–2004)	Cohort	AEDs and epilepsy, *n* = 153 mothers/children No AEDs and no epilepsy, *n* = 236 mothers/children	Valproic acid, carbamazepine, another monotherapy, or polytherapy	Medical records on AED use and dose	Major malformations	Major malformations defined as structural abnormalities requiring medical or surgical intervention to prevent disability using the EUROCAT list of congenital malformations as a guideline
	Dean et al. (1999)	Scotland	Case × control	AEDs, *n* = 46/36/19 (children/mothers/fathers) No AEDs, *n* = 152 adults matched to the parents	Valproic acid, carbamazepine, and phenytoin	The Fetal Anticonvulsant Syndrome Association registry	Fetal anticonvulsant syndrome	Fetal anticonvulsant syndrome in the presence of a characteristic facial appearance and at least one compatible clinical finding (anticonvulsant-associated malformation, medical disorder, or developmental delay), and no other identifiable etiology
**Glucocorticoids**	Van Der Voorn et al. (2015)	The Netherlands; part of the Project On Preterm and Small for gestational age infants (POPS) cohort (1983)	Cohort	Betamethasone, *n* = 71 children No betamethasone, *n* = 273 children	Betamethasone	Medical records	Behavior and intelligence	Behavior assessed by the YASR (self-reported) and YABCL (parent-reported) questionnaires. Intellectual functioning examined with the Multicultural Capacity Test-Intermediate Level (Bleichrodt and van den Berg, 2000)
	Haas et al. (2013)	USA; part of the betamethasone (BMZ) pharmacogenetics cohort	Cohort	Betamethasone, *n* = 109/117 (mothers/children)	Betamethasone	Medical records	Adverse neonatal respiratory outcomes	Neonates with respiratory difficulties included • Neonates needing respiratory support or surfactants (data extracted from neonatal charts) • Neonates diagnosed with bronchopulmonary dysplasia by pediatricians (standard criteria from the Neonatal Research Network of the National Institute of Child Health and Human Development)
	Oretti et al. (2009)	Italy (2005–2006)	Cohort	Betamethasone, *n* = 62 preterm neonates	Betamethasone	Medical records	Respiratory distress syndrome	Diagnosis of respiratory distress syndrome based on clinical and radiographic criteria (tachypnea, chest retractions, cyanosis in room air persisting 48–96 h and typical chest X-ray)
	Bertalan et al. (2008)	Hungary; part of a neonate cohort at Semmelweis University	Cohort	Dexamethasone, *n* = 57 preterm neonates; No dexamethasone, *n* = 68 preterm neonates	Dexamethasone	Medical records	Birth weight and perinatal complications^c^	Medical charts and diagnoses based on clinical and radiographic observations and tests^d^
**Thalidomide**	Kowalski et al. (2020)	Brazil	Case × control	Thalidomide, *n* = 27 affected individuals (*non-Finnish European population of ExAC, 1,000 Genomes, and ABraOM databases were used as a control group for genetic comparisons*)^e^	Thalidomide	Medication use defined based on neonatal phenotype	Thalidomide embryopathy and their specific phenotypes	The authors characterized congenital anomalies related to thalidomide exposure according to a guideline they created. Congenital anomalies were consistent with typical thalidomide embryopathy
	Gomes et al. (2019)	Brazil	Case × control	Thalidomide, *n* = 36 affected individuals; Assumption of no thalidomide by absence of congenital anomalies and thalidomide embryopathy, *n* = 135 individuals	Thalidomide	Medication use defined based on neonatal phenotype	Thalidomide embryopathy and embryopathy-specific phenotypes	The authors characterized congenital anomalies related to thalidomide exposure according to a guideline they created. Congenital anomalies were consistent with typical thalidomide embryopathy
	Gomes et al. (2018)	Brazil	Case × control	Thalidomide, *n* = 35 affected individuals (*non-Finnish European population of gnomAD database was used as a control group*^f^	Thalidomide	Medication use defined based on neonatal phenotype	Thalidomide embryopathy and embryopathy-specific phenotypes	The authors characterized congenital anomalies related to thalidomide exposure according to a guideline they created. Congenital anomalies were consistent with typical thalidomide embryopathy
	Kowalski et al. (2017)	Brazil	Case × control	Thalidomide, *n* = 36 affected individuals Assumption of no thalidomide by absence of congenital anomalies and thalidomide embryopathy, *n* = 136 individuals	Thalidomide	Medication use defined based on neonatal phenotype	Thalidomide embryopathy and embryopathy-specific phenotypes	The authors characterized congenital anomalies related to thalidomide exposure according to a guideline they created. Congenital anomalies were consistent with typical thalidomide embryopathy
	Kowalski et al. (2016)	Brazil	Case × control	Thalidomide, *n* = 38 affected individuals Assumption of no thalidomide by absence of congenital anomalies and thalidomide embryopathy, *n* = 137 individuals	Thalidomide	Medication use defined based on neonatal phenotype	Thalidomide embryopathy and embryopathy-specific phenotypes	The authors characterized congenital anomalies related to thalidomide exposure according to a guideline they created. Congenital anomalies were consistent with typical thalidomide embryopathy
	Vianna et al. (2016)	Brazil	Case × control	Thalidomide, *n* = 38 affected individuals Assumption of no thalidomide by absence of congenital anomalies and thalidomide embryopathy, *n* = 136 individuals	Thalidomide	Medication use defined based on neonatal phenotype	Thalidomide embryopathy and embryopathy-specific phenotypes	The authors characterized congenital anomalies related to thalidomide exposure according to a guideline they created. Congenital anomalies were consistent with typical thalidomide embryopathy
	Vianna et al. (2013)	Brazil	Case × control	Thalidomide, *n* = 28/27 (affected individuals/their relatives) Assumption of no thalidomide by absence of congenital anomalies and thalidomide embryopathy, *n* = 137 individuals	Thalidomide	Medication use defined based on neonatal phenotype	Thalidomide embryopathy and embryopathy-specific phenotypes	The authors characterized congenital anomalies related to thalidomide exposure according to a guideline they created. Congenital anomalies were consistent with typical thalidomide embryopathy
**Other medications**	Bustos et al. (2017)	USA; part of a trial on omega-3 use and spontaneous preterm births	Cohort	17-alpha hydroxyprogesterone caproate, *n* = 268 mothers	17-alpha hydroxy-progesterone caproate	Medical records	17-alpha hydroxy-progesterone caproate plasma concentrations and spontaneous preterm births	17-alpha hydroxyprogesterone caproate plasma concentrations determined by high-performance liquid chromatography-mass spectrometry (limit of detection: 1 ng/mL). Preterm birth based on the gestational age at delivery
	Van Der Zanden et al. (2012)	The Netherlands; subset of the AGORA biobank at Radboud University Nijmegen Medical Center (1980–2008)	Cohort	Estrogens and hypospadias, *n* = 29 children No estrogens and hypospadias, *n* = 580 children	Estrogens	Questionnaires	Hypospadias	Medical records (no information on criteria for diagnosing syndromic hypospadias)
	Perzanowski et al. (2010)	USA (1998–2006)	Cohort	Acetaminophen, *n* = 103 mothers/children No acetaminophen, *n* = 198 mothers/children	Acetaminophen	Questionnaires	Wheeze at 1–3 and 5 years, and seroatopy	Wheeze at 1, 2, or 3 years if ≥1 episode was reported in ≥1 interview during these years. Wheeze at 5 years if an episode was reported in the previous 12 months according to the ISAAC questionnaire. Seroatopy if IgE antibodies in serum ≥0.35 IU/mL against *D. farinae*, mouse, cockroach, cat, or dog
	Shaheen et al. (2010)	Unite Kingdom; Avon Longitudinal Study of Parents and Children (ALSPAC; 1991–1992)	Cohort	Acetaminophen in early pregnancy (< 18–20 weeks), *n* = 4,952 mothers/children Acetaminophen in late pregnancy (20–32 weeks), *n* = 4,822 mothers/children	Acetaminophen	Questionnaires	Wheezing, asthma, eczema, hay fever, atopy, and lung function at 6.5–8.5 years	Children were defined as having wheezing, eczema, and hay fever if mothers responded positively to the question in the interview; asthma was considered based on diagnosis; atopy was defined as a positive reaction to *D. pteronyssinus*, cat, or grass; lung function was measured by spirometry

a *Low birth weight (<2,500 g), small for gestational age, APGAR scores (1 and 5 min) < 7, neonatal breathing problems, neurological symptoms, respiratory distress syndrome, need of access to neonatal intensive care unit, persistent pulmonary hypertension of the neonate, poor neonatal adaptation syndrome; neonatal abstinence syndrome and transient tachypnea of the neonate*.

b *Single heart anomalies, part of complex heart anomalies (including cardiovascular anomalies) or part of complex anomalies involving other organ systems. Diagnosis codes from ICD-9 included 745–746, 7,470–7,474 (excluding 74,550, persistent foramen ovale), and from ICD-10 included Q20–Q26 (excluding Q2111, persistent foramen ovale). These encompass common arterial truncus, transposition of great vessels, single ventricle, ventricular/atrial/atrioventricular septal defects, tetralogy of Fallot, tricuspid atresia and stenosis, Ebstein's anomaly, pulmonary valve stenosis, pulmonary valve atresia, aortic valve atresia/stenosis, hypoplastic left/right heart syndrome, coarctation of aorta, total anomalous pulmonary venous return and patent ductus arteriosus*.

c *Perinatal complications included: necrotizing enterocolitis, intraventricular hemorrhage, patent ductus arteriosus, respiratory distress syndrome, bronchopulmonary dysplasia, and sepsis*.

d *The disorders were diagnosed as follows: necrotizing enterocolitis: clinical and radiological symptoms; intraventricular hemorrhage: neurosonograms; patent ductus arteriosus: echocardiography and clinical signs 5 days after birth; respiratory distress syndrome: need for respiratory support and oxygen upon presence of radiographic chest findings; bronchopulmonary dysplasia: oxygen dependency from 32 weeks of gestational age onwards; sepsis: clinical evidence and/or positive blood/cerebrospinal fluid cultures*.

e *ExAC database, ExAC data is now available in the gnomAD browser; 1,000 Genomes, around 3,000 genomes from individuals of different populations; ABraOM database, 609 exomes from Brazilian individualscultures*.

f *gnomAD database, 125,748 exomes and 15,708 whole genomes from individuals of different populations. ADI-R, Autism Diagnostic Interview-revised; ADOS, autism diagnostic observation schedule; AED, anti-epileptic drug; ASD, autism spectrum disorder; ICD-9/10, International Statistical Classification of Diseases and Related Health Problems 9th/10th Revision; LGD, likely gene-disrupting; SSRI, selective serotonin reuptake inhibitor; SRI, serotonin reuptake inhibitor; YABCL, Young Adult Behavior Checklist; YASR, Young Adult Self Report; TE, thalidomide embryopathy; ISAAC, International Study of Asthma and Allergies in Childhood*.

Several different drugs were investigated, including antidepressants (nine studies), thalidomide (seven studies), antiepileptic drugs (AEDs; five studies), glucocorticoids (four studies), acetaminophen (two studies), and sex hormones (estrogens, one study; 17-alpha hydroxyprogesterone caproate, one study). The main phenotypic outcomes evaluated by the studies were perinatal characteristics and complications (birth weight, Apgar scores, and respiratory distress), protein and neurotransmitter concentrations, congenital anomalies (heart, craniofacial, and limb anomalies, hypospadias, and clefts), and neurodevelopmental outcomes (including child mood, behavior, and executive functions, and disorders such as autism spectrum disorder [ASD]).

An overview of the research methodologies and results of the genetic analyses are presented in Table 2. One study performed whole-exome sequencing, whereas all other studies used a candidate gene approach. Various genotyping methods were applied in the gene candidate studies, and some studies utilized several different approaches. These methods included real-time polymerase chain reaction (real-time PCR; eleven studies), PCR (seven studies), PCR and restriction fragment length polymorphism (RFLP; six studies), genotyping by mass spectrometry (one study), microarray (two studies), Sanger sequencing (three studies), and targeted next-generation sequencing (two studies). DNA was extracted from whole blood (18 studies), cord blood (six studies), placental blood (one study), and/or saliva samples (13 studies) from mothers and/or children. Maternal genetics alone was evaluated in two studies, children's genetics in 14 studies, and both maternal and child genetics in 13 studies. In total, the studies found 48 polymorphisms in 29 genes, as well as several “likely gene-disruptive” mutations, associated with an increased risk of abnormal outcomes in children prenatally exposed to the drugs.

**Table 2:** Overview of pharmacogenetic investigations and results.

	**Reference**	**Sample tissue**	**Approach**	**Method**	**Gene(s) investigated**	**Genetic variants investigated**	**Pharmacogenetic outcomes**
**Antidepressants**	Corti et al. (2019)	Maternal blood and neonatal cord blood samples at birth	Candidate genes	Real-time PCR	*CYP2D6, CYP2C19, CYP2B6, CYP3A4*, and *CYP3A5*	*CYP2D6*: CYP2D6*3, CYP2D6*4, CYP2D6*5, CYP2D6*6, rs1080985 and gene duplication; *CYP2C19*: CYP2C19*2, CYP2C19*3, and CYP2C19*17; *CYP2B6*: CYP2B6*6; *CYP3A4*: CYP3A4*22; *CYP3A5*: CYP3A5*3	• No interaction between the investigated genes and antidepressant exposure on the phenotypic outcomes (*p* > 0.05)
	Ackerman et al. (2017)	Neonatal blood samples	Whole-exome	Whole-exome sequencing	Genes exhibiting *de novo* LGD mutations [4,234 genes with *de novo* mutations identified in Iossifov et al. (2014)]	*De novo* LGD mutations (nonsense, splice site, and frame-shift)	Upon antidepressant exposure: • Children with ASD and **LGD mutations** had increased ADOS-evaluated ASD severity [*F*_(1, 2542)_ = 4.882; *p* = 0.027] • Children with ASD and **LGD mutations** had higher ADI-R verbal communication score [*F*_(1, 2397)_ = 4.554; *p* = 0.033]
	Daud et al. (2017)	Self-sampled buccal cells (swabs) from mother and neonatal	Candidate genes	PCR	*CYP1A2, CYP2C9, CYP2C19, CYP2D6, ABCB1, SLC6A4, HTR1A, HTR1B, HTR2A*, and *HTR3B*	53 variants in the 10 genes investigated^a^	• No interaction found between the investigated genes and antidepressant exposure on the risk of congenital heart anomalies (*p* > 0.05)
	Nembhard et al. (2017)	Self-sampled buccal cells (swabs) from parents and child	Candidate genes	Microarray	60 genes^b^	872 variants in the 60 genes investigated	Upon SSRI exposure: • Increased risk of congenital heart anomalies found if mothers exhibited ***SHMT*** **rs9909104 AA/AG** (*p* = 4.24 × 10^−4^), ***BHMT*** **rs492842 AG/GG** (*p* = 1.23 × 10^−3^), ***BHMT*** **rs542852 AG/GG** (*p* = 2.64 × 10^−3^), or ***MGST1*** **rs2075237 CC/AC** (*p* = 6.97 × 10^−4^)^c^ • Increased risk of congenital heart anomalies found if infants exhibited ***MGMT*** **rs11511217 GG/AG** (*p =* 5.67 × 10^−04^), ***GSTP1*** **rs7941395 GG/AG** (*p =* 1.33 × 10^−3^); ***MGST1*** **rs7294985 GG/AG** (*p =* 9.94 × 10^−4^), ***MTHFS*** **rs1243847 AA/AC** (*p =* 2.44 × 10^−04^), ***TRDMT1*** **rs66022178 AC/CC** (*p =* 3.48 × 10^−3^), or ***GNMT*** **rs11752813 CG/CC** (*p =* 3.71 × 10^−3^)^d^ • Increased risk of obstructive heart defects found if mothers exhibited ***TRDMT1*** **rs2038576 AG/GG** (*p =* 3.39 × 10^−5^) or ***TRDMT1*** **rs2273735 AG/GG** (*p =* 2.33 × 10^−4^)^e^ • Increased risk of obstructive heart defects found if infants exhibited ***TRDMT1*** **rs10795459 AA/AG** (*p =* 2.41 × 10^−4^), ***TRDMT1*** **rs17464824 CC** (*p =* 6.57 × 10^−4^), and ***TRDMT1*** **rs10904893 GG** (*p =* 7. 4 7 × 10^−4^)^f^
	Weikum et al. (2013)	Neonatal whole blood samples	Candidate gene	PCR	*SLC6A4*	*SLC6A4* long and short alleles	• Upon SSRI exposure, children **homozygous for the long allele (ll) of** ***SLC6A4*** had accuracy in the Hearts and Flowers task inversely related to the current severity of maternal depression (β = −0.092; 95% CI−0.171 – −0.014; χ^2^=5.29; *p =* 0.021)
	Brummelte et al. (2013)	Maternal blood and cord blood samples	Candidate gene	PCR	*SLC6A4*	*SLC6A4* long and short alleles	• Upon SRI exposure, mothers **homozygous for the long allele (ll) of** ***SLC6A4*** had greater expression of the 310 kDa reelin band (*p* = 0.007)
	Oberlander et al. (2010)	Maternal blood and cord blood samples	Candidate gene	PCR	*SLC6A4*	*SLC6A4* long and short alleles	• No association of child or maternal *SLC6A4* genotypes with child behavior upon SSRI exposure
	Hilli et al. (2009)	Neonatal blood samples	Candidate genes	Real-time PCR	*SLC6A4, HTR1A, COMT, MAO-A, CYP2C19*, and *CYP2D6*	*SLC6A4*: 5-HTTLPR and 5-HTTVNTR; *HTR1A*: rs6295 and rs6313; *COMT*: rs4680; *MAO-A*: uVNTR (3, 3.5, 4, and 5 copies); *CYP2C19*: CYP2C19*2; *CYP2D6*: CYP2D6*3 and CYP2D6*4	Upon SSRI exposure: • Children with the ***MAO-A*** **genotype 4/4 copies** exhibited higher serotonergic symptom scores (*p* = 0.024) and cord blood dihydroxyphenylglycol concentrations (*p* = 0.0054) • Children with ***COMT*** **rs4680 GG/GA** had higher cord blood prolactin concentrations (*p* = 0.044) • CYP2D6*3 **or** *4 **heterozygous** children had higher fluoxetine concentrations (*p* = 0.033) and active moieties (fluoxetine and norfluoxetine concentrations; *p* = 0.019) in blood
	Oberlander et al. (2008)	Maternal blood and cord blood samples	Candidate gene	PCR	*SLC6A4*	*SLC6A4* long and short alleles	Upon SSRI exposure: • ***SLC6A4*** **heterozygous** neonates had lower birth weight (*F* = 7.45; *p =* 0.008; η^2^ = 0.19) and increased risk of experiencing respiratory distress (*p* = 0.013) • Neonates **homozygous for the long** ***SLC6A4*** **allele** had increased risk of rapid breathing (*p* = 0.018), respiratory distress (*p* = 0.043), and jitteriness (*p* = 0.014) • Neonates **homozygous for the** ***SLC6A4*** **short allele** had higher birth weight (*F* = 4.50; *p* = 0.037; η^2^ = 0.29), lower 5-min Apgar (*p* < 0.001), increased jitteriness (*p* = 0.026), and abnormal tone (*p* = 0.017)
**Antiepileptic drugs**	Jose et al. (2014)	Peripheral blood samples	Candidate genes	PCR and RFLP	*ABCB1, MTHFR, CYP2C9*, and *CYP2C19*	*ABCB1:* rs3213619, rs2214102, rs1202168, rs1128503, rs1922242, rs2032582, and rs1045642; *MTHFR*: rs1801133 and rs1801131; *CYP2C9*: CYP2C9*2 and CYP2C9*3; *CYP2C19*: CYP2C19*2 and CYP2C19*3	Upon AED exposure: • Mothers of children with congenital anomalies had an increased frequency of ***ABCB1*** **rs1202168 CC** (*p* = 0.0032) and the poor metabolizer CYP2C19*2 OR = 1.639 (95% CI 1.150–2.335; *p* = 0.007) and *2*2 genotypes (*p* = 0.005) • Strong association between maternal ***ABCB1*** **GCT haplotype (rs2214102 G allele, rs1202168 C allele, rs1128503 T allele**) and development of congenital anomalies (*p =* 2.18 × 10^−5^)
	Azzato et al. (2010)	Maternal blood samples collected 8 weeks before birth, at birth, and 6 weeks after birth	Candidate genes	PCR	*CYP2C9* and *EPHX1*	*CYP2C9*: CYP2C9*2 and CYP2C9*3; *EPHX1*: rs1051740 and rs2234922	Upon phenytoin exposure: • Craniofacial anomalies were associated with maternal ***EPHX1*** **rs1051740 allele C** (OR = 2.43; 95% CI 1.16–5.10; *p =* 0.018) and **rs2234922 allele G (**OR = 2.33; 95% CI 1.09–5.00; *p =* 0.030) • Maternal ***EPHX1*** **C/G haplotype** (rs1051740/rs2234922) was associated with a risk of cranio-facial anomalies (OR = 6.55; 95% CI 1.37–31.20; *p =* 0.018), whereas the ***EPHX1*** **T/A haplotype** protected against craniofacial anomalies (OR = 0.29; 95% CI 0.12–0.68; *p =* 0.0042)
	Dean et al. (2007)	Parental and neonatal blood or buccal samples	Candidate genes	PCR and RFLP	*MTHFR, SHMT1, MTR, and MTRR*	*MTHFR*: rs1801133 and rs1801131; *SHMT*: rs1979277; *MTR*: rs1805087; *MTRR*: rs1801394	Upon AED exposure: • Maternal ***MTHFR*** **rs1801133 TT** was associated with increased risk of congenital anomalies (RR = 4.09; 95% CI 1.06–15.83; *p =* 0.041) and fetal anticonvulsant syndrome (RR = 3.1; 95% CI 1.09–8.81; *p =* 0.033) compared to the other genotypes • Children with ***MTR*** **rs1805087 GA/GG genotypes** had increased risk of neurodevelopmental delays (OR = 1.76; 95% CI 1.03–3.03; *p =* 0.028) and fetal anticonvulsant syndrome (OR = 1.85; 95% CI 1.13–3.02; *p =* 0.0089) compared to the healthy blood donor controls
	Kini et al. (2007)	Maternal and neonatal buccal samples	Candidate gene	PCR and RFLP	*MTHFR*	rs1801133	• Upon valproic acid exposure (monotherapy or polytherapy), a significantly increased risk of major malformations was associated with the ***MTHFR*** **rs1801133 CT/TT genotypes** compared to controls (non-exposed) CT/TT (OR = 7.79; 95% CI 1.45–41.9; *p*-value not reported)
	Dean et al. (1999)	Parental and neonatal peripheral blood samples	Candidate gene	PCR and RFLP	*MTHFR*	rs1801133	• Mothers of children with fetal anticonvulsant syndrome exposed to AEDs had a higher frequency of ***MTHFR*** **rs1801133 T allele** (*p* < 0.01) and **TT genotype** (*p* < 0.02) compared to mothers of children not exposed to AEDs
**Glucocorticoids**	Van Der Voorn et al. (2015)	Child blood samples	Candidate gene	Genotyping by MS	*NR3C1* and *NR3C2*	*NR3C1*: rs6190 and rs6195; *NR3C2*: rs2070951 and rs5522	Upon betamethasone exposure: • Children carrying ***NR3C1*** **rs6195** variant had a decreased linguistic capacity (*p =* 0.04), ability for logical reasoning (*p =* 0.01), and parent-reported other problems (*p =* 0.03) • Children with ***NR3C2*** **rs2070951 GG** had decreased ability for spatial visualization (*p =* 0.02) and reduced self-reported thought problems (*p =* 0.02) • Children with ***NR3C2*** **rs2070951 CC** had more parent-reported aggressive behavior (*p =* 0.01) • Children carrying ***NR3C2*** **rs5522** had a decreased ability for logical reasoning (*p =* 0.04)
	Haas et al. (2013)	Maternal whole blood or saliva samples and neonatal cord blood or saliva samples	Candidate gene	SNP array, Real-time PCR, and Sanger sequencing	*CYP3A4, CYP3A5, CYP3A7, SULT, ABCB1, NR3C1*, and associated pathway genes^g^	73 SNPs^g^	Upon betamethasone exposure: • Increased risk of bronchopulmonary dysplasia was associated with mothers carrying the minor allele of ***CYP3A7*** **rs113874418** (*p =* 0.006), ***CRHR1*** **Chr. 17-43895531** (*p =* 0.006). or CYP3A7*1E **rs28451617** (OR = 4.97; 95% CI 1.12–22.05; *p =* 0.02), and with child ***NR3C1*** **rs41423247** (OR = 2.56; 95% CI 1.11–5.95; *p =* 0.02) and ***IPO13*** **rs4448553** (OR = 0.01; 95% CI 0.00–0.92; *p =* 0.04) • The need for respiratory support was associated with child ***ABCB1*** **rs1128503 (**OR = 2.23; 95% CI 1.05–4.74; *p =* 0.03) • The need for surfactant was associated with mothers carrying ***IPO13*** **rs2428953** (OR = 13.8; 95 % CI 1.80–105.5; *p =* 0.01) or ***IPO13*** **rs2486014** (OR = 35.5; 95% CI 1.71–736.6; *p =* 0.02) • The need for surfactant replacement therapy was associated with child ***OLR1*** **rs3736233 (**OR = 0.35; 95% CI 0.17–0.71; *p =* 0.003)
	Oretti et al. (2009)	Cord blood samples	Candidate gene	PCR and RFLP	*ABCB1, NR3C1*, and *GST*	*ABCB1*: rs1045642, rs2032582, and rs1128503; *NR3C1*: rs41423247, rs6195, rs6189, and rs6190; *GSTP1*: rs1695; deletions in *GSTM1* and *GSTT1*	• Upon betamethasone exposure, children with respiratory distress syndrome had an increased frequency of the ***GSTP1*** **rs1695 AA genotype** (OR = 3.758; 95% CI 1.16–12.17; *p =* 0.032)
	Bertalan et al. (2008)	Neonatal whole blood or placental blood samples	Candidate gene	PCR and RFLP	*NR3C1*	rs41423247, rs6195, rs6189, and rs6190	• No interaction found between the investigated gene and dexamethasone exposure on the phenotypic outcomes (*p* > 0.05)
**Thalidomide**	Kowalski et al. (2020)	Neonatal saliva samples	Candidate gene	Targeted next generation sequencing	*CRBN, CUL4A, DDB1, IKZF1*, and *IKZF3*	145 variants found across the 5 genes	• Among individuals with thalidomide embryopathy, a higher frequency of ***CRBN*** **rs1045433 G** was found in individuals with pre-axial limb anomalies (*p =* 0.004)
	Gomes et al. (2019)	Neonatal saliva samples	Candidate gene	Targeted next generation sequencing	*ESCO2, TBX5*, and *SALL4*	38 variants found across the 3 genes	• No interaction found between the investigated genes and thalidomide exposure on the susceptibility to thalidomide embryopathy (*p* > 0.05)
	Gomes et al. (2018)	Neonatal saliva samples	Candidate gene	Real-time PCR	*FGF8, FGF10, BMP4, SHH, TP53, TP63*, and *TP73*	*FGF8*: rs1348870; *FGF10*: rs900379; *SHH*: rs28936675; *BMP4*: rs17563; *TP53*: rs1042522; *TP63*: rs17506395; *TP73*: rs2273953	• No interaction found between the investigated genes and thalidomide exposure on the susceptibility to thalidomide embryopathy (*p* > 0.05)
	Kowalski et al. (2017)	Neonatal saliva samples	Candidate gene	Real-time PCR	*NOS2, PTGS2*, and *VEGFA*	*NOS2*: rs2297518*; PTGS2*: rs689465 and rs689466; *VEGFA*: rs3025039, rs1570360, rs2010963, and rs699947	• No interaction found between the investigated genes and thalidomide exposure on the susceptibility to thalidomide embryopathy (*p* > 0.05)
	Kowalski et al. (2016)	Neonatal saliva samples	Candidate gene	Real-time PCR and Sanger sequencing	*NOS3*	rs2070744, rs1799983, and rs61722009	• Individuals with thalidomide embryopathy had a higher frequency of ***NOS3*** **C/4b haplotype** (rs2070744/rs61722009) compared to the control group; the risk of thalidomide embryopathy due to the presence of this haplotype had an OR of 2.57 (95% CI: 1.20–5.80; *p =* 0.018)
	Vianna et al. (2016)	Neonatal saliva samples	Candidate gene	Sanger sequencing	*CRBN* (exons 9–11)	8 non-exonic variants were found	• Among individuals with thalidomide embryopathy, a higher frequency of ***CRBN*** **rs1620675 CC** (100%) was found in individuals with neurological anomalies (*n* = 5) compared to individuals without these anomalies (*p* = 0.004)
	Vianna et al. (2013)	Neonatal saliva samples	Candidate gene	Real-time PCR	*NOS3*	rs2070744 and rs1799983	• Individuals with thalidomide embryopathy had a higher frequency of ***NOS3*** **rs2070744 C allele** (*p* = 0.009) and **CC genotype** (*p* = 0.031) compared to the control group • The control group (with no presumed exposure and no congenital anomalies) had a higher frequency of ***NOS3*** **haplotype T/G** (rs2070744/rs1799983; *p* = 0.004)
**Other medications**	Bustos et al. (2017)	Maternal blood samples	Candidate gene	Real-time PCR	*CYP3A4, CYP3A5*, and *NR3C3*	*CYP3A4*: *r*s35599367, rs2242480, and rs2740574; *CYP3A5*: rs776746; *NR3C3*: rs578029, rs471767, rs666553, rs503362 rs500760	• No interaction found between the investigated genes and 7-alpha hydroxyprogesterone caproate exposure on 17-alpha hydroxyprogesterone caproate plasma concentrations or spontaneous preterm births (*p* > 0.05)
	Van Der Zanden et al. (2012)	Child blood samples	Candidate gene	Real-time PCR	*SRD5A2, ESR1, ESR2*, and *ATF3*	*SRD5A2*: rs523349; *ESR1*: rs6932902; *ESR2*: rs2987983; *ATF3*: rs11119982	• Upon estrogen exposure, the risk of hypospadias was significantly associated with the presence of ***SRD5A2*** **rs523349 CG (**RR = 2.7; 95% CI 1.0–7.4) or **GG genotypes** (RR = 2.9; 95% CI 1.0–8.3) in the affected individuals (*p* < 0.001)
	Perzanowski et al. (2010)	Maternal and neonatal peripheral blood samples	Candidate gene	Real-time PCR and PCR	*GSTP1, GSTT2, GSTT1*, and *GSTM1*	*GSTP1*: rs1695; *GSTT2*: rs2719; *GSTT1* and *GSTM1* gene deletion	Upon acetaminophen exposure: • Children with ***GSTP1*** **rs1695 AG/GG genotypes** had increased risk of current wheeze (RR = 2.08; 95% CI 1.34–3.23; *p* = 0.001) and seroatopy at 5 years (RR = 1.96; 95% CI 1.36–2.84; *p* < 0.001) • There was evidence of effect modification in the presence of at least one allele of ***GSTT1*** in seroatopy (RERI = 0.98; 95% CI 0.50–1.5; *p* < 0.001)
	Shaheen et al. (2010)	Maternal whole blood or saliva samples	Candidate gene	Real-time PCR	*NRF2, GSTP1, GSTT1*, and *GSTM1*	*NRF2*: rs6706649; *GSTP1*: rs1695; *GSTT1* and *GSTM1* gene deletion	Upon acetaminophen exposure in early pregnancy: • Mothers with ***NRF2*** **rs6706649 TC/TT genotypes** had increased risk of having children with asthma (OR = 1.73; 95% CI 1.22–2.45; *p* = 0.002) and wheezing (OR = 1.53; 95% CI 1.06–2.20; *p* = 0.024) • Mothers with at least on allele of ***GSTT1*** (OR = 1.24; 95% CI 1.02–1.50; *p* = 0.03) or two copies of alleles of ***GSTM1*** (OR = 1.96; 95% CI 1.09–3.51; *p* = 0.03) had increased risk of having children with asthma Upon acetaminophen exposure in late pregnancy: • Mothers with ***NRF2*** **rs6706649 TC/TT genotypes** had increased risk of having children with asthma (OR = 1.63; 95% CI 1.13–2.37; *p* = 0.009) • Mothers with at least on allele of ***GSTT1*** had increased risk of having children with asthma (OR = 1.39; 95% CI 1.14–1.70; *p* = 0.001) and wheezing (OR = 1.27; 95% CI 1.03–1.56; *p* = 0.03), and mothers with at least one allele of ***GSTM1*** had increased risk of having children with asthma (OR = 1.41; 95% CI 1.02–1.95; *p* = 0.04) or wheezing (OR = 1.60; 95% CI 1.15–2.24; *p* = 0.006)

*The bolded values in the tables indicate the significant genes and genotypes discovered in each study*.

a *CYP1A2: rs2069521, rs2069526, rs4646425, rs4646427, rs2472304, rs2470890; CYP2C9: rs1799853, rs1057910, rs56165452, rs9332131, rs28371686, rs7900194, rs28371685, rs72558187, rs72558190; CYP2C19: rs4244285, rs4986893, rs28399504, rs56337013, rs72552267, rs72558186, rs41291556, rs17884712, rs6413438, rs12248560; CYP2D6: rs16947, rs35742686, rs5030867, rs5030656, rs1065852, rs5030863, rs5030862; ABCB1: rs1128503, rs2032582, rs1045642, rs2235040, rs4148739, rs1882478, rs9282564, rs10256836; SLC6A4: rs4795541, rs57098334; HTR1A: rs1364043, rs6295; HTR1B: rs6296, rs6298; HTR2A: rs7997012, rs6313, rs6314, rs1928040, rs6311; HTR3B: rs1176744, rs3831455;*

b *Genes not reported;*

c *Mothers: SHMT rs9909104, AG RR = 2.43 (1.46–4.03) or GG RR = 5.90 (2.13–16.24); BHMT rs492842, AG RR = 2.15 (1.33–3.46) or GG RR = 4.62 (1.77–11.97); BHMT rs542852, AG RR = 2.06 (1.26–3.34); MGST1 rs2075237, CC RR = 7.95 (2.50–25.40) or AC RR = 2.82 (1.58–5.04);*

d *Children: MGMT rs11511217, AG RR = 2.41 (1.45–4.01); GSTP1 rs7941395, GG RR = 4.71 (1.90–11.63) or AG RR = 2.17 (1.38–3.41); MGST1 rs7294985, GG RR = 6.10 (0.10–17.72) or AG RR = 2.47 (1.45–4.21); MTHFS rs1243847, AA RR = 6.76 (2.56–17.89) or AC RR = 2.60 (1.60–4.23); TRDMT1 rs66022178, AC RR = 1.79 (1.14–2.80) or CC RR = 3.2 (1.30–7.84); GNMT rs11752813, CG RR = 1.80 (1.15–2.80) or CC RR = 3.24 (1.32–7.84);*

e *Mothers: TRDMT rs2038576, AG RR = 4.64 (2.20–9.77) or GG RR = 21.53 (4.84–95.45); TRDMT rs2273735, AG RR = 3.20 (1.67–6.10) or GG RR = 10.24 (2.79–37.21);*

f *Children: TRDMT1 rs10795459, AA RR = 9.00 (2.66–30.36) or AG RR = 3.0 (1.63–5.51); TRDMT1 rs17464824, CC RR = 6.81 (2.07–22.56) or AC RR = 2.61 (1.44–4.75); TRDMT1 rs10904893, GG RR = 6.97 (2.13–22.47) or AG RR = 2.64 (1.46–4.74);*

g ABCB1: rs1128503, rs2032582, rs1045642; ADCY9: rs2230739; CRH: rs12721511; CRHR1: rs1876828, rs242941; CYP3A4: rs4987159; CYP3A5*3: rs776746; CYP3A7: rs2687133; CYP3A7*2: rs2257401; GLCCI1: rs37973; IPO13: rs7412307, rs2301992, rs2301993, rs2428953, rs4448553, rs2486014, rs2240447, rs1990150, rs6671164, rs1636879; NR3C1: rs41423247; OLR1: rs3736233; STIP1/FERMT3: rs2236647; SULT1A2: rs1136703; TBX21: rs2240017; TRAPPC5/FCER2: rs28364072; CRHR1: rs4564621, rs2316763, Chr. 17- 43895531, rs242939, rs2316764, rs4277389, rs6658607; Chr. 17- 43895671, rs80249281, rs11316767, rs116593005, rs149144271, rs8077279, rs16940655; CYP3A7: rs45496695, rs45446698, rs113874418, rs11568826, rs45467892, rs45575938, rs45494802, rs11568824; CYP3A7*1B: rs45465393; CYP3A7*1C: rs11568825; CYP3A7*1D: rs55798860; CYP3A7*1E: rs28451617; NR3C1: rs6189, rs6190, rs72542738, rs72481829, rs61759025, rs79138720, rs6192, rs72558023, rs72542745, rs72558022, rs13306589, rs56149945, rs1800445; CYP3A4*1B: rs2740574; CYP3A5*6: rs10264272; CYP3A5*7: rs41303343; SULT1A1*2: rs9282861; SULT1A1: rs3760091, rs750155. AED, anti-epileptic drug; ASD, autism spectrum disorder; ADI-R, Autism Diagnostic Interview-revised; ADOS, autism diagnostic observation schedule; CI, confidence interval; LGD, likely gene-disruptive; MS, mass spectrometry; OR, odds ratio; PCR, polymerase chain reaction; RERI, relative excess risk due to interaction; RFLP, restriction fragment length polymorphism; RR, risk ratio; SNP, single nucleotide polymorphism; SRI, serotonin reuptake inhibitor; SSRI, selective serotonin reuptake inhibitor.

### Genetic Susceptibility Associated With Drug Teratogenicity

The main outcomes regarding genetic teratology are presented below. The genes referred to in the following paragraphs are described with their full name and function in Appendix 4.

#### Antidepressants

Antidepressants, in particular serotonin reuptake inhibitors (SRIs) examined in eight out of nine studies, was the most investigated class of drug in the literature on genetic variations associated with adverse drug effects *in utero*. *SLC6A4*, which encodes the serotonin transporter, was a commonly investigated gene in studies on genetic vulnerability for a wide range of different antidepressants and phenotypic outcomes. Variants of *SLC6A4* was associated with executive function accuracy in children (Weikum et al., 2013), blood reelin levels related to neonatal sleeping disturbances and irritability (Brummelte et al., 2013), and perinatal characteristics, such as birth weight, 5-min Apgar score, muscular tone, and respiratory complications (Oberlander et al., 2008). The *MAO-A, COMT*, and *CYP2D6* genotypes were also associated with adverse outcomes upon antidepressant exposure, with genetic variants of these genes associated with serotonergic symptoms and abnormal neurotransmitter concentrations in infants (Hilli et al., 2009). Furthermore, genetic variants of *SHMT1, BHMT, MGST1, MGMT, MTHFS, GNMT*, and *TRDMT1* were associated with an increased risk of congenital heart defects after prenatal antidepressant exposure (Nembhard et al., 2017). Finally, in individuals with ASD prenatally exposed to antidepressants, the presence of “likely gene-disruptive” mutations was associated with increased severity of symptoms of ASD (Ackerman et al., 2017).

#### Thalidomide

Thalidomide was the main individually investigated drug. All studies have been performed by the same research group in the approximately same sample of individuals (minor changes in sample sizes depending on the study). In these studies, the *CRBN* and *NOS3*, which are part of the CLR4-Cereblon complex, and act on angiogenic and oxidative stress processes, respectively, were reported to be associated with an increased risk of developing thalidomide embryopathy, a phenotypic spectrum of malformations characterized by limb reduction defects (Vargesson, 2009). The *CRBN* genetic variants rs1045433 and rs1620675 were associated with an increased risk of pre-axial limb anomalies and neurological anomalies, respectively (Vianna et al., 2016; Kowalski et al., 2020). In addition, *NOS3* has deleterious variants (rs2070744, rs1799983, and rs61722009) associated with susceptibility to developing thalidomide embryopathy (Vianna et al., 2013; Kowalski et al., 2016).

#### Antiepileptic Drugs

*MTHFR*, associated with folate metabolism, was the most frequently investigated gene in studies on the genetic susceptibility to AED teratogenesis. The most commonly studied AEDs were valproic acid, carbamazepine, and phenytoin. Upon AED exposure, the maternal genetic variant rs1801133 of *MTHFR* was associated with an increased risk of major malformations and fetal anticonvulsant syndrome in the children (Dean et al., 1999, 2007; Kini et al., 2007). Another genetic variant investigated in the AED studies was the rs1805087 variant of *MTR* in children, which was associated with an increased risk of neurodevelopmental delay and fetal anticonvulsant syndrome in AED-exposed children (Dean et al., 2007). The maternal variants CYP2C19^*^2 and rs1202168 of *ABCB1*, as well as the haplotype G/C/T (rs2214102/rs1202168/rs1128503) of *ABCB1*, were also associated with an increased risk of congenital anomalies upon AED exposure (Jose et al., 2014). One study specifically examined the genetic susceptibility to phenytoin teratogenicity and found that a maternal haplotype of *EPHX1* C/G (rs1051740/rs2234922) is associated with an increased risk of offspring craniofacial anomalies (Azzato et al., 2010).

#### Glucocorticoids

All studies on glucocorticoid exposure investigated *NR3C1*, the gene encoding the glucocorticoid receptor, as a susceptibility factor for glucocorticoid teratogenesis. Neonates exposed to glucocorticoids *in utero* and carriers of the genetic variant *NR3C1* rs6195 have poorer language and cognitive abilities (Van Der Voorn et al., 2015) and an increased risk of bronchopulmonary dysplasia (Haas et al., 2013). Another gene associated with glucocorticoid adverse outcomes is *NR3C2*, which encodes the mineralocorticoid receptor. Specific child genotypes (genetic variants rs2070951 and rs5522) were associated with decreased abilities in spatial visualization and logical reasoning, and increased aggressive behavior (Van Der Voorn et al., 2015). In addition, the *CYP3A7* (rs113874418 and rs28451617), *CRHR1* (Chr. 17-43895531), *ABCB1* (rs1128503), *OLR1* (rs3736233), *IPO13* (rs2428953 and rs2486014), and *GSTP1* (rs1695) genotypes in children were associated with respiratory complications upon glucocorticoid exposure (Haas et al., 2013).

#### Acetaminophen

Two studies investigated prenatal acetaminophen exposure (Perzanowski et al., 2010; Shaheen et al., 2010). The study by Perzanowski et al. (2010) found an association between the genetic variant rs1695 in *GSTP1* and increased risk of wheezing at 5 years of age as well as association between the presence of at least one allele of GSTT1 and increased risk of seroatopy, in children exposed to acetaminophen during pregnancy. Both of these genes encode glutathione S-transferases (GSTs). The other study, by Shaheen et al. (2010), also found an association of the presence of at least one allele of *GSTT1* in mothers with an increased risk of children presenting with wheezing or asthma at 6.5–7.5 years of age. This association was also demonstrated for the maternal variant *NRF2* rs6706649 and for the presence in the mother of at least one allele of *GSTM1*.

#### Sex Hormones

In the one study investigating prenatal estrogen exposure, the genetic variant rs523349 located in *SRD5A2* was associated with an increased risk of hypospadias (Van Der Zanden et al., 2012). Bustos et al. (2017) investigated the potential effect of *CYP3A4, CYP3A5*, and *NR3C3* on the plasma concentrations of 7-alpha hydroxyprogesterone caproate (a progestin) and the interaction of these genes with the drug in spontaneous premature delivery. However, the authors did not find any gene-outcome associations (Bustos et al., 2017).

## Discussion

### Key Findings and Methodological Limitations

Teratogenesis is a complex event in which different factors affect the outcome in an individual. Knowledge of the teratogenic potential of many drugs is still limited and disputed, and no unique list of likely teratogenic drugs exists. In contrast to Wilson's other principles of teratogenesis (Wilson, 1977), the evaluation of genetic susceptibility to teratogens represents a challenge in regards to both research methodologies and strategies, and the interpretation and application of results. Therefore, we performed a systematic review to summarize and compare the findings of studies investigating the association of the mother and/or child's genetic background with the risk of congenital anomalies or developmental abnormalities due to prenatal exposure to drugs with teratogenic potential.

In studies investigating SRI exposure, *SLC6A4*, which encodes the serotonin transporter, was the gene most commonly associated with adverse fetal outcomes. Serotonin has several important roles during embryonic and fetal brain development, including neuronal maturation, migration, synaptogenesis, and differentiation of neural crest cells associated with facial and cardiac development (Brummelte et al., 2017). Brummelte et al. (2017) suggest that these important roles of serotonin may explain why SSRIs and variations in *SLC6A4* could impair embryonic and fetal development.

Thalidomide is one of the oldest known teratogenic agents, causing thalidomide embryopathy (Vargesson, 2009). The reviewed studies on thalidomide focused on examining genes encoding proteins important in angiogenesis and oxidative stress, or proteins that are part of the CLR4-Cereblon complex, all of which are known to be associated with thalidomide teratogenicity (Therapontos et al., 2009; Ito et al., 2010; Vianna et al., 2013, 2016; Ha et al., 2016; Kowalski et al., 2016, 2017, 2020).

The reviewed studies on AEDs mainly investigated *MTHFR*, which is associated with folate metabolism (Dean et al., 1999, 2007; Kini et al., 2007; Jose et al., 2014). Folate is essential for both methylation and DNA synthesis, and folate metabolism is important for normal fetal development (Nazki et al., 2014). It has been demonstrated that AED treatment is associated with lower mean serum folate levels, but the mechanisms underlying this association remain speculative and might include interferences with diet, absorption, plasma binding, cellular metabolism, and renal excretion (Linnebank et al., 2011). Therefore, examining functional polymorphisms in genes of proteins involved in the folate pathway could be highly relevant to better understanding the differential susceptibility of individuals to the teratogenic effects of AEDs.

Glucocorticoids are usually related to the stimulation of fetal growth and organ maturation. However, an excess of synthetic glucocorticoids can negatively affect neonatal development (Clifton et al., 2017). All of the reviewed studies on glucocorticoid exposure investigated *NR3C1*, which encodes the glucocorticoid receptor, as a susceptibility factor for glucocorticoid teratogenesis. As glucocorticoids act via this receptor, the presence of genetic variants that change the glucocorticoid binding site could affect the biological functioning of the receptor in fetal development (Adcock and Mumby, 2016; Busada and Cidlowski, 2017).

The two studies investigating the genetic susceptibility to acetaminophen teratogenesis found that genes encoding glutathione S-transferases (GSTs) are associated with the presence of child wheezing and asthma (Perzanowski et al., 2010; Shaheen et al., 2010). GSTs are involved in acetaminophen pharmacokinetics and are important for the prevention of lung damage through the detoxification of reactive oxygen species generated by inflammation, infection, and other exposures (Zhao and Pickering, 2011). Therefore, the effects of acetaminophen exposure on respiratory outcomes have been suggested to be modified by GST activity (Dai et al., 2020).

The use of estrogens and other hormones during pregnancy has been associated with male reproductive disorders, such as hypospadias and cryptorchidism (Storgaard et al., 2006). This observation was replicated by Van Der Zanden et al. (2012), who found that *SRD5A2* (variant rs523349) is associated with hypospadias upon prenatal estrogen exposure.

Although a large number of drugs were included in our list of search terms (Appendices 1, 2), only a few of these drugs were investigated in prenatal pharmacogenetic studies, even among the drugs with known teratogenic properties. In particular, of the twelve known teratogenic drugs in Appendix 1, nine were not investigated in either of the reviewed studies (including ACE inhibitors, antineoplastic agents, diethylstilbestrol, fingolimod, lithium, misoprostol, mycophenolate, retinoids, and warfarin). Another limitation of most of the studies in this review was a small sample size. In addition, some studies re-used the samples across multiple projects, which can decrease the power for finding strong and reliable associations. Small sample sizes also limit the power to study medications at a substance level. This is unfortunate as even small variations in chemical structure may impact the embryo-fetal toxicity of the drug. For instance, four out of the five reviewed studies on AEDs, were performed on medications with important structural, pharmacological and teratogenic differences, such as lamotrigine (Mufson, 2018) and valproic acid (Rosenberg, 2007). Group-level examinations may be acceptable if the medications within the specific group are expected to exhibit similar mechanisms of action and similar embryo-fetal toxicity (e.g., SSRIs). Furthermore, most studies used a candidate gene approach, whereas only one study applied a genome-wide approach (Ackerman et al., 2017). Candidate gene approaches limit the comparison of results and the application of the results in future studies due to the *a priori* selection of a few genes. This may be an appropriate approach if there is a strong hypothesis of certain genes being involved but, in most cases, current knowledge is too sparse to support this as the preferred approach. Therefore, comprehensive genome-wide association studies (GWASs) may considerably broaden the search for potentially interesting genes.

### Future Directions: Toward Personalized Medicine

To realize the potential of studies within genetic teratology, it is important to work toward overcoming current limitations and to improve our approaches within this field. Such efforts may ultimately enable clinical implementation of prenatal pharmacogenetic screening. This will enhance the clinical relevance and drive the field toward personalized medicine for pregnant women in need of drugs.

Teratogenesis is a complex, multifactorial process and an understanding of both genetic and environmental factors is pivotal to predicting the effects of a teratogen (Kowalski et al., 2020). Furthermore, multiple genetic variants with small individual effects are likely to be associated with teratogenic susceptibility (Edwards et al., 2013). Upon exposure to a teratogen, the synergistic effect of the genetic and pharmaceutical factors may cause teratogenesis. It is essential to perform genome-wide investigations to account for the multiple genetic variants associated with a phenotypic outcome of prenatal drug exposure. If only a candidate gene approach is feasible, the study should have a strong hypothesis for selecting the gene(s). Such a rationale may be founded on biological plausibility derived from animal and *in vitro* studies, or known involvement of the genes in drug metabolism.

Investigation of complex teratogenic processes, likely involving non-linear and non-additive interactions of multiple genetic and environmental factors, are further supported by the development of novel methods within bioinformatics (Weber et al., 2019). Analyzing and deciphering such higher-order interactions prompts the application of machine learning models, which can detect patterns and predict drug teratogenicity and child outcomes. For example, machine learning has been applied in the investigation of non-teratogenic birth defect profiles, predicting groups at high risk of congenital heart defects (Luo et al., 2017), drugs of great concern during pregnancy (Ailes et al., 2020), and the probability of a drug being teratogenic (Challa et al., 2020).

Health authorities and funding bodies play a pivotal role in facilitating further advances in research on genetic teratology. Such institutions should take on the responsibility of supporting research, particularly for drugs that have had conflicting results, such as antidepressants and AEDs, but also ADHD, antithyroid, and HIV drugs. Support can be provided by making more research funding available, such as through initiatives like the IMI ConcePTION project (ConcePTION, 2020). In addition, health authorities should consider requesting pharmacogenetic studies from the market authorization holders if gene polymorphisms are expected to play a significant role in drug teratogenicity.

Finally, genetic profiling of human fetuses is essential to translate findings from studies on genetic teratology into clinical diagnostics. However, access to tissue specimens during pregnancy is often limited due to the risk of affecting normal development or causing miscarriages by invasive sampling techniques (Salomon et al., 2019). Therefore, it is important that novel methods, such as non-invasive prenatal testing (NIPT; Lau et al., 2012; Allyse et al., 2015), are developed and applied clinically. Moreover, developing and implementing personalized medicine approaches like those described above in clinical practice, will require multidisciplinary teams with clinical geneticists, bioinformaticians, obstetricians, and reproductive toxicologists.

## Conclusion

In summary, this systematic review identified 29 studies on human genetic variation associated with the teratogenesis of several drugs. Antidepressants, AEDs, glucocorticoids, and thalidomide were the most investigated drugs. The most convincing findings were genetic variants in *SLC6A4, MTHFR*, and *NR3C1* being associated with drug teratogenicity from antidepressants, AEDs, and glucocorticoids, respectively. Future studies on genetic teratology should aim to consider the biological pathways and molecular mechanisms of teratogens more broadly, and take into consideration the complexity of the multifactorial teratogenic process. This approach requires increased sample sizes, the application of genome-wide approaches, and international funding initiatives. To translate the research into the clinic, application of novel methodologies for the collection and examination of fetal DNA is encouraged, as well as the use of machine learning methods and implementation of multidisciplinary teams. Taken together, these efforts may ultimately promote early interventions and possibly minimize the teratogenic impact of maternal drug use during pregnancy on the fetus.

## Data Availability Statement

The original contributions presented in the study are included in the article/Supplementary Material, further inquiries can be directed to the corresponding author/s.

## Author Contributions

JAG, EWO, LS-F, and HMEN contributed to the study conception and design. JAG and EWO performed the literature searches and data analyses. JAG wrote the manuscript. All authors critically revised the manuscript.

## Conflict of Interest

The authors declare that the research was conducted in the absence of any commercial or financial relationships that could be construed as a potential conflict of interest.
