# Supplementary material for: Genetic Susceptibility to Drug Teratogenicity: A Systematic Literature Review
Source: Front Genet. 2021 Apr 27;12:645555. doi: 10.3389/fgene.2021.645555 (PMC8107476; doi:10.3389/fgene.2021.645555)
Supplement: Supplementary file 1 [file Data_Sheet_1.docx]

**Supplementary Material**

| **Appendix 1.** List of major human teratogens with evidence of human teratogenicity or fetotoxicity. |
| --- |
| 1. ACE inhibitors (e.g., captopril, enalapril, lisinopril, and ramipril) |
| 1. Antineoplastic agents (e.g., cisplatin, cyclophosphamide, and doxorubicin) |
| 1. Diethylstilbestrol |
| 1. Fingolimod |
| 1. Lithium |
| 1. Misoprostol |
| 1. Mycophenolic acid/mycophenolate |
| 1. Phenytoin |
| 1. Retinoids (e.g., alitretinoin, isotretinoin, and tretinoin) |
| 1. Thalidomide and its analogues pomalidomide and lenalidomide |
| 1. Valproic acid |
| 1. Warfarin |

**References:**

Schaefer, C. P. P. W. J. . M. R. K. (2014). *Drugs During Pregnancy and Lactation - 3rd Edition*. 3rd ed. Academic Press.

| **Appendix 2.** Concerns about risk of human teratogenicity or fetotoxicity with conflicting or weak evidence.   \| 1. Adrenergic beta-antagonists (e.g., atenolol, labetalol, propranolol) \| \| --- \| \| 1. Analgesics (e.g., opiates such as morphine and codeine) \| \| 1. Androgens (e.g., danazol) \| \| 1. Antidepressive agents (e.g., SSRIs such as paroxetine, SNRIs such as venlafaxine, and TCAs such as amitriptyline) \| \| 1. Anticonvulsants (e.g., barbiturates, carbamazepine, primidone, topiramate, and trimethadione) \| \| 1. Antifungal/antiprotozoals agents (e.g., azoles such as ketoconazole and metronidazole) \| \| 1. Anti-infective agents (e.g., aminoglycosides, sulfonamides, and tetracyclines) \| \| 1. Antimalarials (e.g., chloroquine, mefloquine, and quinine) \| \| 1. Antimetabolities (e.g., fluorouracil, mercaptopurine, methotrexate, and thioguanine) \| \| 1. Antiviral agents (e.g., dolutegravir and zidovudine) \| \| 1. AT1-receptor antagonists (e.g., irbesartan, losartan, and valsartan) \| \| 1. Antipsychotic agents (e.g., haloperidol and quetiapine) \| \| 1. Benzodiazepines (e.g., alprazolam, luvasta, clonazepam, and lorazepam) \| \| 1. Bisphosphonates (e.g., alendronate and zoledronic acid) \| \| 1. Central nervous system stimulants (e.g., methylphenidate and modafinil) \| \| 1. Endothelin receptor antagonists (e.g., ambrisentan, bosentan, and macitentan) \| \| 1. Enzyme replacement therapy (e.g., alglucosidase alpha and taliglucerase alpha) \| \| 1. Ergot alkaloids (e.g., ergotamine) \| \| 1. Estrogens (e.g., estradiol) \| \| 1. Glucocorticoids (e.g., cortisone and prednisone) \| \| 1. Immunosuppressive agents (e.g., leflunomide) \| \| 1. Monoclonal antibodies (e.g., eculizumab and rituximab) \| \| 1. NSAIDs – non-selective or COX-2 inhibitors (e.g., aspirin, celecoxib, and diclofenac) \| \| 1. Oral contraceptives (e.g., ethinylestradiol) \| \| 1. Progestins (e.g., medroxyprogesterone and norethindrone) \| \| 1. Selective estrogen receptor modulators (e.g., clomiphene) \| \| 1. Serotonin 5-HT3 receptor antagonists (e.g., ondansetron) \| \| 1. Statins (e.g., atorvastatin, rosuvastatin, and simvastatin) \| \| 1. Thioamides (e.g., methimazole and propylthiouracil) \| |
| --- | --- | --- | --- | --- | --- | --- | --- | --- | --- | --- | --- | --- | --- | --- | --- | --- | --- | --- | --- | --- | --- | --- | --- | --- | --- | --- | --- | --- | --- |

SSRI: selective serotonin reuptake inhibitor; SNRI: serotonin-noradrenaline reuptake inhibitor; TCA: tricyclic antidepressant; NSAID: non-steroidal anti-inflammatory drug.

**References:**

Altıntaş Aykan D, Ergün Y. Hormonal Contraceptives: What if Exposed During Pregnancy? Erciyes Med J 2019; 41(1): 50-5.

Alwan S, Polifka JE, Friedman JM. Angiotensin II receptor antagonist treatment during pregnancy. Birth Defects Res A Clin Mol Teratol. 2005; 73(2):123-30.

Bánhidy F, Ács N, Puhó E, Czeizel AE. Ergotamine treatment during pregnancy and a higher rate of low birthweight and preterm birth. J Clin Pharmacol. 2007; 64(4): 510–516.

Bergman JEH, Lutke LR, Gans ROB, Addor MC, Barisic I, *et al.* Beta-Blocker Use in Pregnancy and Risk of Specific Congenital Anomalies: A European Case-Malformed Control Study. Drug Saf. 2018; 41(4): 415–427.

Bolea-Alamanac BM, Green A, Verma G, Maxwell P, Davies SJ. Methylphenidate use in pregnancy and lactation: a systematic review of evidence. Br J Clin Pharmacol. 201;77(1):96-101.

Bookstaver PB, Bland CM, Griffin B, Stover KR, Eiland LS, McLaughlin M. A Review of Antibiotic Use in Pregnancy. Pharmacotherapy. 2015; 35(11):1052-62.

Brunskill PJ. The effects of fetal exposure to danazol. Br J Obstet Gynaecol. 1992; 99(3):212-5.

Bullo M, Tschumi S, Bucher BS, Bianchetti MG, Simonetti GD. Pregnancy outcome following exposure to angiotensin-converting enzyme inhibitors or angiotensin receptor antagonists: a systematic review. Hypertension. 2012; 60(2):444-50.

Byatt N, Deligiannidis KM, Freeman MP. Antidepressant use in pregnancy: a critical review focused on risks and controversies. Acta Psychiatr Scand. 2013; 127(2):94-114.

Charlton B, Mølgaard-Nielsen D, Svanström H, Wohlfahrt J, Pasternak B, Melbye M. Maternal use of oral contraceptives and risk of birth defects in Denmark: prospective, nationwide cohort study. BMJ 2016; 352:h6712.

Clementi M, Di Gianantonio E, Cassina M, Leoncini E, Botto LD, Mastroiacovo P; SAFE-Med Study Group. Treatment of hyperthyroidism in pregnancy and birth defects. J Clin Endocrinol Metab. 2010; 95(11):E337-41.

de Raaf MA, Beekhuijzen M, Guignabert C, Vonk Noordegraaf A, Bogaard HJ. Endothelin-1 receptor antagonists in fetal development and pulmonary arterial hypertension. Reprod Toxicol. 2015; 56:45-51.

Schaefer, C. P. P. W. J. . M. R. K. (2014). *Drugs During Pregnancy and Lactation - 3rd Edition*. 3rd ed. Academic Press.

Duan L, Ng A, Chen W, Spencer HT, Nguyen J, Shen AY, Lee MS.
β-Blocker Exposure in Pregnancy and Risk of Fetal Cardiac Anomalies. JAMA Intern Med. 2017; 177(6):885-887.

Enato E, Moretti M, Koren G. The fetal safety of benzodiazepines: an updated meta-analysis. J Obstet Gynaecol Can. 2011; 33(1):46-48.

Giannubilo SR, Pasculli A, Tidu E, Ciavattini A. Replacement Therapy for Gaucher Disease during Pregnancy: A Case Report. J Reprod Infertil. 2015; 16(1):53-7.

Hemminki E, Gissler M, Toukomaa H. Exposure to female hormone drugs during pregnancy: effect on malformations and cancer. Br J Cancer. 1999; 80(7):1092-7.

Hill DS, Wlodarczyk BJ, Palacios AM, Finnell RH. Teratogenic effects of antiepileptic drugs Expert Rev Neurother. 2010; 10(6): 943–959.

Huybrechts KF, Hernández-Díaz S, Patorno E, Desai RJ, Mogun H, Dejene SZ, Cohen JM, Panchaud A, Cohen L, Bateman BT. Antipsychotic Use in Pregnancy and the Risk for Congenital Malformations. JAMA Psychiatry. 2016; 73(9):938-46.

Interrante JD, Ailes EC, Lind JN, Anderka M, Feldkamp ML, Werler MM, Taylor LG, Trinidad J, Gilboa SM, Broussard CS; National Birth Defects Prevention Study. Risk comparison for prenatal use of analgesics and selected birth defects, National Birth Defects Prevention Study 1997-2011. Ann Epidemiol. 2017; 27(10): 645–653.e2.

Iqbal MM, Aneja A, Rahman A, Megna J, Freemont W, Shiplo M, Nihilani N, Lee K. The potential risks of commonly prescribed antipsychotics: during pregnancy and lactation. Psychiatry (Edgmont). 2005; 2(8):36-44.

Källén B, Borg N, Reis M. The Use of Central Nervous System Active Drugs During Pregnancy. Pharmaceuticals 2013; 1221-1286.

Karalis DG, Hill AN, Clifton S, Wild RA. The risks of statin use in pregnancy: A systematic review. J Clin Lipidol. 2016; 10(5):1081-90.

Kemp MW, Newnham JP, Challis JG, Jobe AH, Stock SJ. The clinical use of corticosteroids in pregnancy. Hum Reprod Update. 2016; 22(2):240-59.

Li X, Liu GY, Ma JL, Zhou L. Risk of congenital anomalies associated with antithyroid treatment during pregnancy: a meta-analysis. Clinics (Sao Paulo). 2015; 70(6):453-9.

Lunghi L, Pavan B, Biondi C, Paolillo R, Valerio A, Vesce F, Patella A. Use of glucocorticoids in pregnancy. Curr Pharm Des. 2010; 16(32):3616-37.

Lupattelli A, Chambers CD, Bandoli G, Handal M, Skurtveit S, Nordeng H. Association of Maternal Use of Benzodiazepines and Z-Hypnotics During Pregnancy With Motor and Communication Skills and Attention-Deficit/Hyperactivity Disorder Symptoms in Preschoolers. JAMA Netw Open. 2019; 2(4):e191435.

Nezvalová-Henriksen K, Spigset O, Nordeng H. Effects of ibuprofen, diclofenac, naproxen, and piroxicam on the course of pregnancy and pregnancy outcome: a prospective cohort study. BJOG. 2013; 120(8):948-59.

Oliveira Santos M, Evangelista T, Conceição I. Enzyme replacement therapy with alglucosidase alfa in a late-onset Pompe disease patient during pregnancy. Neuromuscul Disord. 2018; 28(11):965-968.

Pasternak B, Svanström H, Hviid A. Ondansetron in pregnancy and risk of adverse fetal outcomes. N Engl J Med. 2013; 368(9):814-23.

Ponticelli C, Moroni G. Fetal Toxicity of Immunosuppressive Drugs in Pregnancy J. Clin. Med. 2018: 7:552.

Scaparrotta A, Chiarelli F, Verrotti A. Potential Teratogenic Effects of Clomiphene Citrate. Drug Saf. 2017; 40(9):761-769.

Selig BP, Furr JR, Huey RW, Moran C, Alluri VN, Medders GR, Mumm CD, Hallford HG, Mulvihill JJ. Cancer chemotherapeutic agents as human teratogens. Postgrad Med. 2010 Jul;122(4):49-65.

Sokal A, Elefant E, Leturcq T, Beghin D, Mariette X, Seror R. Pregnancy and newborn outcomes after exposure to bisphosphonates: a case-control study. Osteoporos Int. 2019; 30(1):221-229.

Tagbor H, Antwi G, Dogbe J. Safety of antimalarial drugs exposure during early pregnancy. Research and Reports in Tropical Medicine. 2014; 5:23-33.

Tuccori M, Montagnani S, Testi A, Ruggiero E, Mantarro S, Scollo C, Pergola A, Fornai M, Antonioli L, Colucci R, Corona T, Blandizzi C. Use of selective serotonin reuptake inhibitors during pregnancy and risk of major and cardiovascular malformations: an update. Birth Defects Res A Clin Mol Teratol. 2012; 94(8):626-50.

van Gelder MM, de Jong-van den Berg LT, Roeleveld N. Drugs associated with teratogenic mechanisms. Part II: a literature review of the evidence on human risks. Hum Reprod. 2014; 29(1):168-83.

van Gelder MM, Roeleveld N, Nordeng H. Exposure to non-steroidal anti-inflammatory drugs during pregnancy and the risk of selected birth defects: a prospective cohort study.  PLoS One. 2011; 6(7):e22174.

van Gelder MM, van Rooij IALM, Miller RK, Zielhuis GA, Jong-van den Berg LTW, Roeleveld N. Teratogenic mechanisms of medical drugs. Human Reproduction Update. 2010; 16:378–394.

Ward SA, Sevene EJ, Hastings IM, Nosten F, McGready R. Antimalarial drugs and pregnancy: safety, pharmacokinetics, and pharmacovigilance. The Lancet Infectious Diseases. 2007; 7(2):136–144.

**Appendix 3: Search Terms**

**PubMed**

| **Drug* Or drug classes** | Angiotensin-Converting Enzyme Inhibitors[Mh] OR Angiotensin-Converting Enzyme Inhibitor*[Tiab] OR ACE Inhibitor*[Tiab] OR Angiotensin Converting Enzyme Antagonist*[Tiab] OR Angiotensin I Converting Enzyme Inhibitor*[Tiab] OR Kininase II Antagonist*[Tiab] OR Kininase II Inhibitor*[Tiab] OR Antineoplastic Agents[Mh] OR Antineoplastic Agent*[Tiab] OR Antineoplastic Drug*[Tiab] OR Antineoplastic*[Tiab] OR Antitumor Drug*[Tiab] OR Anticancer Agent*[Tiab] OR Antitumor Agent*[Tiab] OR Diethylstilbestrol[Mh] OR diethylstilbestrol[Tiab] OR Stilbene Estrogen[Tiab] OR Apstil[Tiab] OR Stilbestrol[Tiab] OR Distilbène[Tiab] OR Tampovagan[Tiab] OR Agostilben[Tiab] OR Fingolimod Hydrochloride[Mh] OR Fingolimod Hydrochloride[Tiab] OR Gilenya[Tiab] OR Gilenia[Tiab] OR Fingolimod[Tiab] OR Lithium[Mh] OR lithium[Tiab] OR Misoprostol[Mh] OR misoprostol[Tiab] OR Novo-Misoprostol[Tiab] OR Novo Misoprostol[Tiab] OR Apo-Misoprostol[Tiab] OR Apo Misoprostol[Tiab] OR Glefos[Tiab] OR Cytotec[Tiab] OR Mycophenolic Acid[Mh] OR mycophenolic Acid[Tiab] OR Mycophenolate Mofetil[Tiab] OR Mycophenolate Sodium[Tiab] OR Cellcept[Tiab] OR Sodium Mycophenolate[Tiab] OR Myfortic[Tiab] OR Mycophenolate Mofetil Hydrochloride[Tiab] OR Phenytoin[Mh] OR phenytoin[Tiab] OR Fenitoin[Tiab] OR Diphenylhydantoin[Tiab] OR Difenin[Tiab] OR Dihydan[Tiab] OR Phenytoin Sodium[Tiab] OR Sodium Diphenylhydantoinate[Tiab] OR Epamin[Tiab] OR Epanutin[Tiab] OR Hydantol[Tiab] OR Antisacer[Tiab] OR Dilantin[Tiab] OR Retinoids[Mh] OR Retinoid*[Tiab] OR Thalidomide[Mh] OR thalidomide[Tiab] OR Thalomid[Tiab] OR Sedoval[Tiab] OR Pomalidomide[Mh] OR pomalidomide[Tiab] OR Imnovid[Tiab] OR Pomalyst[Tiab] OR Lenalidomide[Mh] OR lenalidomide[Tiab] OR Revlimid[Tiab] OR Valproic acid[Mh] OR valproic acid[Tiab] OR 2-Propylpentanoic Acid[Tiab] OR 2 Propylpentanoic Acid[Tiab] OR Divalproex[Tiab] OR Depakene[Tiab] OR Convulsofin[Tiab] OR Depakote[Tiab] OR Dipropyl Acetate[Tiab] OR Divalproex Sodium[Tiab] OR Semisodium Valproate[Tiab] OR Vupral[Tiab] OR Propylisopropylacetic Acid[Tiab] OR Ergenyl[Tiab] OR Magnesium Valproate[Tiab] OR Valproate[Tiab] OR Valproate Sodium[Tiab] OR Sodium Valproate[Tiab] OR Calcium Valproate[Tiab] OR Valproate Calcium[Tiab] OR Depakine[Tiab] OR Warfarin[Mh] OR warfarin[Tiab] OR Apo-Warfarin[Tiab] OR Aldocumar[Tiab] OR Gen-Warfarin[Tiab] OR Warfant[Tiab] OR Coumadin[Tiab] OR Marevan[Tiab] OR Warfarin Potassium[Tiab] OR Potassium, Warfarin[Tiab] OR Warfarin Sodium[Tiab] OR Sodium, Warfarin[Tiab] OR Coumadine[Tiab] OR Tedicumar[Tiab] OR Adrenergic beta-antagonists[Mh] OR Adrenergic beta-antagonist*[Tiab] OR Adrenergic beta Antagonist*[Tiab] OR beta-Adrenoceptor Antagonist*[Tiab] OR beta Adrenoceptor Antagonist*[Tiab] OR Adrenergic beta-Blocker*[Tiab] OR beta-Adrenergic Receptor Blockader*[Tiab] OR beta Adrenergic Receptor Blockader*[Tiab] OR beta-Adrenergic Blocking Agent*[Tiab] OR Agents, beta-Adrenergic Blocking[Tiab] OR beta Adrenergic Blocking Agent*[Tiab] OR beta-Adrenergic Blocker*[Tiab] OR beta Adrenergic Blocker*[Tiab] OR beta-Adrenergic Antagonist*[Tiab] OR beta Adrenergic Antagonist*[Tiab] OR Adrenergic beta-Receptor Blockader*[Tiab] OR Adrenergic beta Receptor Blockader*[Tiab] OR Analgesics[Mh] OR Analgesic*[Tiab] OR Analgesic Drug*[Tiab] OR Anodyne*[Tiab] OR Analgesic Agent*[Tiab] OR Antinociceptive Agent*[Tiab] OR Androgens[Mh] OR Androgen*[Tiab] OR Androgenic Compound*[Tiab] OR Androgenic Agent*[Tiab] OR Androgen Effect[Tiab] OR Androgen Effect*[Tiab] OR Antidepressive Agents[Mh] OR Antidepressive Agent*[Tiab] OR Antidepressant Drug*[Tiab] OR Antidepressant*[Tiab] OR Thymoanaleptic*[Tiab] OR Thymoleptic*[Tiab] OR Serotonin Uptake Inhibitors[Mh] OR Serotonin Uptake Inhibitor*[Tiab] OR Serotonin Reuptake Inhibitor*[Tiab] OR Selective Serotonin Reuptake Inhibitor*[Tiab] OR SSRI*[Tiab] OR Serotonin and Noradrenaline Reuptake Inhibitors[Mh] OR Serotonin and Noradrenaline Reuptake Inhibitor*[Tiab] OR SSRIs and NRI*[Tiab] OR NRIs and SSRI*[Tiab] OR Serotonin and Noradrenaline Uptake Inhibitor*[Tiab] OR SNRI*[Tiab] OR Serotonin and Norepinephrine Reuptake Inhibitor*[Tiab] OR Serotonin and Norepinephrine Uptake Inhibitor*[Tiab] OR Antidepressive Agents, Tricyclic[Mh] OR Antidepressive Agents, Tricyclic[Tiab] OR Tricyclic Antidepressive Agent*[Tiab] OR Tricyclic Antidepressant Drug*[Tiab] OR Tricyclic Antidepressant*[Tiab] OR Anticonvulsants[Mh] OR Anticonvulsant*[Tiab] OR Anticonvulsive Agent*[Tiab] OR Anticonvulsive Drug*[Tiab] OR Anticonvulsant Drug*[Tiab] OR Antiepileptic Agent*[Tiab] OR Antiepileptic*[Tiab] OR Antiepileptic Drug*[Tiab] OR Antifungal Agents[Mh] OR Antifungal Agent*[Tiab] OR Therapeutic Fungicide*[Tiab] OR Antifungal Antibiotic*[Tiab] OR Antiprotozoal Agents[Mh] OR Antiprotozoal Agent*[Tiab] OR Schizonticide*[Tiab] OR Anti-Infective Agents[Mh] OR Anti-Infective Agent*[Tiab] OR Anti Infective Agent*[Tiab] OR Antiinfective Agent*[Tiab] OR Microbicide*[Tiab] OR Antimicrobial Agent*[Tiab] OR Anti-Microbial Agent*[Tiab] OR Anti Microbial Agent*[Tiab] OR Antimalarials[Mh] OR Antimalarial*[Tiab] OR Antimalarial Agent*[Tiab] OR Antimalarial Drug*[Tiab] OR Anti-Malarial*[Tiab] OR Anti Malarial*[Tiab] OR Antimetabolites[Mh] OR Antimetabolite*[Tiab] OR Antineoplastic Antimetabolite*[Tiab] OR Antiviral Agents[Mh] OR Antiviral Agent*[Tiab] OR Antiviral*[Tiab] OR Antiviral Drug*[Tiab] OR Angiotensin II Type 1 Receptor Blockers[Mh] OR Angiotensin II Type 1 Receptor Blocker*[Tiab] OR Angiotensin II Type 1 Receptor Antagonist*[Tiab] OR Type 1 Angiotensin Receptor Blocker*[Tiab] OR Angiotensin 2 Type 1 Receptor Antagonist*[Tiab] OR Type 1 Angiotensin Receptor Antagonist*[Tiab] OR Selective Angiotensin II Receptor Antagonist*[Tiab] OR Antipsychotic agents[Mh] OR Antipsychotic agent*[Tiab] OR Antipsychotic*[Tiab] OR Major Tranquilizer*[Tiab] OR Major Tranquillizing Agent*[Tiab] OR Neuroleptic Drug*[Tiab] OR Neuroleptic*[Tiab] OR Major Tranquilizing Agent*[Tiab] OR Antipsychotic Drug*[Tiab] OR Neuroleptic Agent*[Tiab] OR Antipsychotic Effect[Tiab] OR Antipsychotic Effect*[Tiab] OR Benzodiazepines[Mh] OR Benzodiazepine*[Tiab] OR Benzodiazepine[Tiab] OR Benzodiazepine Compound*[Tiab] OR Bisphosphonate*[Tiab] OR Central Nervous System Stimulants[Mh] OR Central Nervous System Stimulant*[Tiab] OR Central Stimulant*[Tiab] OR CNS Stimulant*[Tiab] OR Analeptic*[Tiab] OR Analeptic Drug*[Tiab] OR Analeptic Agent*[Tiab] OR Endothelin receptor antagonists[Mh] OR Endothelin receptor antagonist*[Tiab] OR Endothelin Antagonist*[Tiab] OR Enzyme Replacement Therapy[Mh] OR Enzyme Replacement Therapy[Tiab] OR Enzyme Replacement Therap*[Tiab] OR Ergot Alkaloids[Mh] OR Ergot Alkaloid*[Tiab] OR Ergotamine[Tiab] OR Estrogens[Mh] OR Estrogen*[Tiab] OR Estrogenic Compound*[Tiab] OR Estrogenic Agent*[Tiab] OR Estrogen[Tiab] OR Estrogen Receptor Agonist*[Tiab] OR Estrogen Effect[Tiab] OR Estrogenic Effect[Tiab] OR Estrogenic Effect*[Tiab] OR Estrogen Effect*[Tiab] OR Glucocorticoids[Mh] OR Glucocorticoid*[Tiab] OR Glucocorticoid[Tiab] OR Glucocorticoid Effect[Tiab] OR Glucorticoid Effect*[Tiab] OR Immunosuppressive agents[Mh] OR Immunosuppressive agent*[Tiab] OR Immunosuppressant*[Tiab] OR Monoclonal Antibod*[Tiab] OR NSAID*[Tiab] OR Non-Steroidal Anti-Inflammatory Agent*[Tiab] OR Non-Steroidal Anti Inflammatory Agent*[Tiab] OR NonSteroidal Anti-Inflammatory Agent*[Tiab] OR NonSteroidal Antiinflammatory Agent*[Tiab] OR Anti-Inflammatory Analgesic*[Tiab] OR Aspirin-Like Agent*[Tiab] OR Aspirin Like Agent*[Tiab] OR Oral Contraceptive*[Tiab] OR Phasic Oral Contraceptive*[Tiab] OR Low-Dose Oral Contraceptive*[Tiab] OR Progestins[Mh] OR Progestin*[Tiab] OR Progestagen*[Tiab] OR Progestational Hormone*[Tiab] OR Progestogen*[Tiab] OR Gestagen[Tiab] OR Progestational Compound*[Tiab] OR Progestagenic Agent*[Tiab] OR Gestagenic Agent*[Tiab] OR Progestin[Tiab] OR Gestagen*[Tiab] OR Progestational Agent*[Tiab] OR Progestin Effect[Tiab] OR Progestogen Effect[Tiab] OR Gestagenic Effect*[Tiab] OR Gestagen Effect[Tiab] OR Gestagen Effect*[Tiab] OR Gestagenic Effect[Tiab] OR Progestin Effect*[Tiab] OR Progestogen Effect*[Tiab] OR Selective Estrogen Receptor Modulator[Mh] OR Selective Estrogen Receptor Modulator[Tiab] OR SERM*[Tiab] OR Selective Estrogen Receptor Modulator[Tiab] OR SERM[Tiab] OR Serotonin 5-HT3 Receptor Antagonists[Mh] OR Serotonin 5-HT3 Receptor Antagonist*[Tiab] OR Serotonin 5 HT3 Receptor Antagonist*[Tiab] OR 5-HT3 Antagonist[Tiab] OR 5 HT3 Antagonist[Tiab] OR 5-HT3 Antagonist*[Tiab] OR 5 HT3 Antagonist*[Tiab] OR Hydroxymethylglutaryl-CoA Reductase Inhibitors[Mh] OR Hydroxymethylglutaryl-CoA Reductase Inhibitor*[Tiab] OR Hydroxymethylglutaryl CoA Reductase Inhibitor*[Tiab] OR HMG-CoA Reductase Inhibitor*[Tiab] OR HMG CoA Reductase Inhibitor*[Tiab] OR HMG-CoA Statin*[Tiab] OR Hydroxymethylglutaryl-CoA Inhibitor*[Tiab] OR Statin*[Tiab] OR Hydroxymethylglutaryl-Coenzyme A Inhibitor*[Tiab] OR Thioamides[Mh] OR Thioamide*[Tiab] |
| --- | --- |
| **Teratogenic effects** | Teratogenesis[Mh] OR Teratogenesis[Tiab] OR teratogen[Tiab] OR teratogens[Tiab] OR Congenital Abnormalities[Mh] OR Congenital Abnormalities[Tiab] OR Congenital Abnormality[Tiab] OR Deformities[Tiab] OR Deformity[Tiab] OR Congenital Defects[Tiab] OR Congenital Defect[Tiab] OR Birth Defects[Tiab] OR Birth Defect[Tiab] OR congenital anomalies[Tiab] OR congenital anomaly[Tiab] OR Cardiovascular Abnormalities[Mh] OR Cardiovascular Abnormalities[Tiab] OR Cardiovascular Abnormalities[Tiab] OR Eye Abnormalities[Mh] OR Eye Abnormalities[Tiab] OR Eye Abnormalities[Tiab] OR Congenital Microtia[Mh] OR Congenital Microtia[Tiab] OR Congenital Microtias[Tiab] OR Anotia[Tiab] OR Anotias[Tiab] OR Microtia[Tiab] OR Microtias[Tiab] OR Musculoskeletal Abnormalities[Mh] OR Musculoskeletal Abnormalities[Tiab] OR Musculoskeletal Abnormality[Tiab] OR Craniofacial Abnormalities[Mh] OR Craniofacial Abnormalities[Tiab] OR Craniofacial Abnormality[Tiab] OR Nervous System Malformations[Mh] OR Nervous System Malformations[Tiab] OR Nervous System Malformation[Tiab] OR Nervous System Abnormalities[Tiab] OR Nervous System Abnormality[Tiab] OR Nervous System Anomalies[Tiab] OR Nervous System Anomaly[Tiab] OR Nervous System Congenital Abnormalities[Tiab] OR Nervous System Congenital Abnormality[Tiab] OR Respiratory System Abnormalities[Mh] OR Respiratory System Abnormalities[Tiab] OR Respiratory System Abnormalities[Tiab] OR Urogenital Abnormalities[Mh] OR Urogenital Abnormalities[Tiab] OR Urogenital Abnormality[Tiab] OR Genitourinary Abnormalities[Tiab] OR Genitourinary Abnormality[Tiab] OR Congenital Disorders[Tiab] OR Congenital Disorder[Tiab] OR Neonatal Abnormalities[Tiab] OR Neonatal Abnormality[Tiab] OR Fetal Diseases[Mh] OR Fetal Diseases[Tiab] OR Fetal Disease[Tiab] OR Embryopathy[Tiab] OR Embryopathies[Tiab] OR Digestive System Abnormalities[Mh] OR Digestive System Abnormalities[Tiab] OR Digestive System Abnormality[Tiab] OR Abnormalities, Drug-Induced[Mh] OR Drug Induced Abnormalities[Tiab] OR Drug-Induced Abnormalities[Tiab] OR Limb Deformities, Congenital[Mh] OR Limb Deformities, Congenital[Tiab] OR Congenital Limb Deformities[Tiab] OR Congenital Limb Deformity[Tiab] OR Polydactyly[Mh] OR Polydactyly[Tiab] OR Polydactylia[Tiab] OR Polydactylias[Tiab] OR Syndactyly[Mh] OR Syndactyly[Tiab] OR Syndactylia[Tiab] OR Syndactylias[Tiab] OR Autism Spectrum Disorder[Mh] OR Autism Spectrum Disorder[Tiab] OR Autism Spectrum Disorders[Tiab] OR Communication Disorders[Mh] OR Communication Disorders[Tiab] OR Communication Disorder[Tiab] OR Communicative Disorders[Tiab] OR Communicative Disorder[Tiab] OR Developmental Communication Disorder[Tiab] OR Developmental Communication Disorders[Tiab] OR Communication Disabilities[Tiab] OR Communication Disability[Tiab] OR Intellectual Disability[Mh] OR Intellectual Disability[Tiab] OR Intellectual Disabilities[Tiab] OR Intellectual Development Disorder[Tiab] OR Intellectual Development Disorders[Tiab] OR Mental Retardation[Tiab] OR Psychosocial Mental Retardation[Tiab] OR Psychosocial Mental Retardations[Tiab] OR Mental Deficiencies[Tiab] OR Mental Deficiency[Tiab] OR Learning Disabilities[Mh] OR Learning Disabilities[Tiab] OR Learning Disability[Tiab] OR Low-Birth-Weight Infant[Tiab] OR Low Birth Weight Infant[Tiab] OR Low Birth Weight Infants[Tiab] OR Low-Birth-Weight Infants[Tiab] OR Low Birth Weight[Tiab] OR Low Birth Weights[Tiab] OR Attention Deficit Disorder with Hyperactivity[Mh] OR Attention Deficit Disorder with Hyperactivity[Tiab] OR Attention Deficit Disorders with Hyperactivity[Tiab] OR Attention Deficit Hyperactivity Disorders[Tiab] OR Attention Deficit-Hyperactivity Disorder[Tiab] OR Attention Deficit-Hyperactivity Disorders[Tiab] OR Hyperkinetic Syndrome[Tiab] OR ADDH[Tiab] OR Attention Deficit Hyperactivity Disorder[Tiab] OR Attention Deficit Disorder[Tiab] OR Attention Deficit Disorders[Tiab] OR Minimal Brain Dysfunction[Tiab] OR Developmental Disabilities[Mh] OR Developmental Disabilities[Tiab] OR Developmental Disability[Tiab] OR Child Development Disorder[Tiab] OR Child Development Disorders[Tiab] OR Developmental Delay Disorders[Tiab] OR Developmental Delay Disorder[Tiab] OR Child Development Deviations[Tiab] OR Child Development Deviation[Tiab] OR Language Development Disorders[Mh] OR Language Development Disorders[Tiab] OR Language Development Disorder[Tiab] OR Developmental Language Disorders[Tiab] OR Developmental Language Disorder[Tiab] OR Speech Delay[Tiab] OR Speech Delays[Tiab] OR Semantic-Pragmatic Disorder[Tiab] OR Semantic Pragmatic Disorder[Tiab] OR Semantic-Pragmatic Disorders[Tiab] OR Central Auditory Processing Disorder[Tiab] OR Language Delay[Tiab] OR Language Delays[Tiab] OR Infant, Small for Gestational Age[Mh] OR Infant, Small for Gestational Age[Tiab] OR Motor Skills Disorders[Mh] OR Motor Skills Disorders[Tiab] OR Motor Skills Disorder[Tiab] OR Developmental Coordination Disorders[Tiab] OR Developmental Coordination Disorder[Tiab] OR Fertility[Mh] OR Fertility[Tiab] OR Fecundity[Tiab] OR Mutagenity[Tiab] OR Reproductive toxicity[Tiab] OR Developmental toxicity[Tiab] OR Embryonic Development[Mh] OR Embryonic Development [Tiab] OR Embryonic Developments[Tiab] OR Embryogenesis[Tiab] OR Embryo Development[Tiab] OR Embryonic Programming[Tiab] OR Embryonic Programmings[Tiab] OR Postimplantation Embryo Development[Tiab] OR Post-implantation Embryo Development[Tiab] OR Post implantation Embryo Development[Tiab] OR Embryonic and Fetal Development[Mh] OR Embryo and Fetal Development[Tiab] OR Prenatal Exposure Delayed Effects[Mh] OR Prenatal Exposure Delayed Effects[Tiab] OR perinatal toxicity[Tiab] OR postnatal toxicity[Tiab] OR peri and postnatal toxicity[Tiab] OR Fetal Development[Mh] OR Fetal Development[Tiab] OR Fetal Growth[Tiab] OR embryo toxicity[Tiab] OR embryotoxicity[Tiab] OR fetal toxicity[Tiab] OR fetaltoxicity[Tiab] OR embryofetal toxicity[Tiab] OR developmental toxicity[Tiab] OR development toxicity[Tiab] |
| **Genetic Predisposition** | Genetic Predisposition to Disease[Mh] OR Genetic Predisposition to Disease[Tiab] OR Genetic Susceptibility[Tiab] OR Genetic Susceptibilities[Tiab] OR Genetic Predisposition[Tiab] OR Genetic Predispositions[Tiab] OR polymorphism[Tiab] OR polymorphisms[Tiab] OR Genetic Variation[Mh] OR Genetic Variation[Tiab] OR Genetic Variations[Tiab] OR Genetic Diversities[Tiab] OR Genetic Diversity[Tiab] OR Polymorphism, Genetic[Mh] OR Polymorphism, Genetic[Tiab] OR Genetic Polymorphisms[Tiab] OR Genetic Polymorphism[Tiab] |

**EMBASE**

| **Drug or drug classes** | exp *dipeptidyl carboxypeptidase i inhibitor/ OR exp *Antineoplastic agent/ OR exp *carbamazepine/ OR exp *diethylstilbestrol/ OR exp *fingolimod/ OR exp *lithium/ OR exp *misoprostol/ OR exp *mycophenolic acid/ OR exp *phenytoin/ OR exp *Retinoid/ OR exp *thalidomide/ OR exp *pomalidomide/ OR exp *lenalidomide/ OR exp *valproic acid/ OR exp *warfarin/ OR exp *beta adrenergic receptor blocking agent/ OR exp *analgesic agent/OR exp *Androgen/ OR exp *antidepressant agent/ OR exp *serotonin uptake inhibitor/ OR exp *noradrenalin uptake Inhibitor/ OR exp *anticonvulsive agent/ OR exp *antifungal agent/ OR exp *antiprotozoal agent/ OR exp *Anti-infective agent/ OR exp *antimalarial agent/ OR exp *Antimetabolite/ OR exp *antivirus agent/ OR exp *angiotensin 1 receptor antagonist/ OR exp *neuroleptic agent/ OR exp *benzodiazepine derivative/ OR exp * bisphosphonic acid derivative/ OR exp *central stimulant agent/ OR exp *endothelin receptor antagonist/ OR exp *enzyme replacement/ OR exp *ergot alkaloid/ OR exp *Estrogen/ OR exp *glucocorticoid/ OR exp *Immunosuppressive agent/ OR exp *monoclonal antibody/ OR exp *nonsteroid antiinflammatory agent/ OR exp *oral contraceptive agent/ OR exp *gestagen/ OR exp *selective estrogen receptor modulator/ OR exp *serotonin 3 antagonist/ OR exp *hydroxymethylglutaryl coenzyme A reductase inhibitor/ OR exp *thioamide/ OR (Angiotensin-Converting Enzyme Inhibitor* OR ACE Inhibitor* OR Angiotensin Converting Enzyme Antagonist* OR Angiotensin I Converting Enzyme Inhibitor* OR Kininase II Antagonist* OR Kininase II Inhibitor* OR Antineoplastic Agent* OR Antineoplastic Drug* OR Antineoplastic* OR Antitumor Drug* OR Anticancer Agent* OR Antitumor Agent* OR carbamazepine OR Carbamazepine Acetate OR Carbamazepine Dihydrate OR Carbamazepine Hydrochloride OR Carbamazepine L-Tartrate OR Carbamazepine Anhydrou* OR Carbamazepine Sulfate OR Carbazepin OR Epitol OR Finlepsin OR Neurotol OR Tegretol OR Amizepine OR Carbamazepine Phosphate OR diethylstilbestrol OR Stilbene Estrogen OR Apstil OR Stilbestrol OR Distilbene OR Tampovagan OR Agostilben OR Fingolimod Hydrochloride OR Gilenya OR Gilenia OR Fingolimod OR lithium OR misoprostol OR Novo-Misoprostol OR Novo Misoprostol OR Apo-Misoprostol OR Apo Misoprostol OR Glefo* OR Cytotec OR mycophenolic Acid OR Mycophenolate Mofetil OR Mycophenolate Sodium OR Cellcept OR Sodium Mycophenolate OR Myfortic OR Mycophenolate Mofetil Hydrochloride OR phenytoin OR Fenitoin OR Diphenylhydantoin OR Difenin OR Dihydan OR Phenytoin Sodium OR Sodium Diphenylhydantoinate OR Epamin OR Epanutin OR Hydantol OR Antisacer OR Dilantin OR Retinoid* OR thalidomide OR Thalomid OR Sedoval OR pomalidomide OR Imnovid OR Pomalyst OR lenalidomide OR Revlimid OR valproic acid OR 2-Propylpentanoic Acid OR 2 Propylpentanoic Acid OR Divalproex OR Depakene OR Convulsofin OR Depakote OR Dipropyl Acetate OR Divalproex Sodium OR Semisodium Valproate OR Vupral OR Propylisopropylacetic Acid OR Ergenyl OR Magnesium Valproate OR Valproate OR Valproate Sodium OR Sodium Valproate OR Calcium Valproate OR Valproate Calcium OR Depakine OR Warfarin OR Apo-Warfarin OR Aldocumar OR Gen-Warfarin OR Warfant OR Coumadin OR Marevan OR Warfarin Potassium OR Warfarin Sodium OR Coumadine OR Tedicumar OR Adrenergic beta-antagonist* OR Adrenergic beta Antagonist* OR beta-Adrenoceptor Antagonist* OR beta Adrenoceptor Antagonist* OR Adrenergic beta-Blocker* OR beta-Adrenergic Receptor Blockader* OR beta Adrenergic Receptor Blockader* OR beta-Adrenergic Blocking Agent* OR Agents, beta-Adrenergic Blocking OR beta Adrenergic Blocking Agent* OR beta-Adrenergic Blocker* OR beta Adrenergic Blocker* OR beta-Adrenergic Antagonist* OR beta Adrenergic Antagonist* OR Adrenergic beta-Receptor Blockader* OR Adrenergic beta Receptor Blockader* OR Analgesic* OR Analgesic Drug* OR Anodyne* OR Analgesic Agent* OR Antinociceptive Agent* OR Androgen* OR Androgenic Compound* OR Androgenic Agent* OR Androgen Effect OR Androgen Effect* OR Antidepressive Agent* OR Antidepressant Drug* OR Antidepressant* OR Thymoanaleptic* OR Thymoleptic* OR Serotonin Uptake Inhibitor* OR Serotonin Reuptake Inhibitor* OR Selective Serotonin Reuptake Inhibitor* OR SSRI* OR Serotonin and Noradrenaline Reuptake Inhibitor* OR SSRIs and NRI* OR NRIs and SSRI* OR Serotonin and Noradrenaline Uptake Inhibitor* OR SNRI* OR Serotonin and Norepinephrine Reuptake Inhibitor* OR Serotonin and Norepinephrine Uptake Inhibitor* OR Antidepressive Agents, Tricyclic OR Tricyclic Antidepressive Agent* OR Tricyclic Antidepressant Drug* OR Tricyclic Antidepressant* OR Anticonvulsant* OR Anticonvulsive Agent* OR Anticonvulsive Drug* OR Anticonvulsant Drug* OR Antiepileptic Agent* OR Antiepileptic* OR Antiepileptic Drug* OR Antifungal Agent* OR Therapeutic Fungicide* OR Antifungal Antibiotic* OR Antiprotozoal Agent* OR Schizonticide* OR Anti-Infective Agent* OR Anti Infective Agent* OR Antiinfective Agent* OR Microbicide* OR Antimicrobial Agent* OR Anti-Microbial Agent* OR Anti Microbial Agent* OR Antimalarial* OR Antimalarial Agent* OR Antimalarial Drug* OR Anti-Malarial* OR Anti Malarial* OR Antimetabolite* OR Antineoplastic Antimetabolite* OR Antiviral Agent* OR Antiviral* OR Antiviral Drug* OR Angiotensin II Type 1 Receptor Blocker* OR Angiotensin II Type 1 Receptor Antagonist* OR Type 1 Angiotensin Receptor Blocker* OR Angiotensin 2 Type 1 Receptor Antagonist* OR Type 1 Angiotensin Receptor Antagonist* OR Selective Angiotensin II Receptor Antagonist* OR Antipsychotic agent* OR Antipsychotic* OR Major Tranquilizer* OR Major Tranquillizing Agent* OR Neuroleptic Drug* OR Neuroleptic* OR Major Tranquilizing Agent* OR Antipsychotic Drug* OR Neuroleptic Agent* OR Antipsychotic Effect OR Antipsychotic Effect* OR Benzodiazepine* OR Benzodiazepine OR Benzodiazepine Compound* OR Bisphosphonate* OR Central Nervous System Stimulant* OR Central Stimulant* OR CNS Stimulant* OR Analeptic* OR Analeptic Drug* OR Analeptic Agent* OR Endothelin receptor antagonist* OR Endothelin Antagonist* OR Replacement Therapy OR Enzyme Replacement Therap* OR Ergot Alkaloid* OR Ergotamine OR Estrogen* OR Estrogenic Compound* OR Estrogenic Agent* OR Estrogen OR Estrogen Receptor Agonist* OR Estrogen Effect OR Estrogenic Effect OR Estrogenic Effect* OR Estrogen Effect* OR Glucocorticoid* OR Glucocorticoid OR Glucocorticoid Effect OR Glucorticoid Effect* OR Immunosuppressive agent* OR Immunosuppressant* OR Monoclonal Antibod* OR NSAID* OR Non-Steroidal Anti-Inflammatory Agent* OR Non-Steroidal Anti Inflammatory Agent* OR Nonsteroidal Anti-Inflammatory Agent* OR Nonsteroidal Antiinflammatory Agent* OR Anti-Inflammatory Analgesic* OR Aspirin-Like Agent* OR Aspirin Like Agent* OR Oral Contraceptive* OR Phasic Oral Contraceptive* OR Low-Dose Oral Contraceptive* OR Progestin* OR Progestagen* OR Progestational Hormone* OR Progestogen* OR Gestagen OR Progestational Compound* OR Progestagenic Agent* OR Gestagenic Agent* OR Progestin OR Gestagen* OR Progestational Agent* OR Progestin Effect OR Progestogen Effect OR Gestagenic Effect* OR Gestagen Effect OR Gestagen Effect* OR Gestagenic Effect OR Progestin Effect* OR Progestogen Effect* OR Selective Estrogen Receptor Modulator OR SERM* OR Selective Estrogen Receptor Modulator OR SERM OR Serotonin 5-HT3 Receptor Antagonist* OR Serotonin 5 HT3 Receptor Antagonist* OR 5-HT3 Antagonist OR 5 HT3 Antagonist OR 5-HT3 Antagonist* OR 5 HT3 Antagonist* OR Hydroxymethylglutaryl-CoA Reductase Inhibitor* OR Hydroxymethylglutaryl CoA Reductase Inhibitor* OR HMG-CoA Reductase Inhibitor* OR HMG CoA Reductase Inhibitor* OR HMG-CoA Statin* OR Hydroxymethylglutaryl-CoA Inhibitor* OR Statin* OR Hydroxymethylglutaryl-Coenzyme A Inhibitor* OR Thioamide*).tw,kw,dy. |
| --- | --- |
| **Teratogenic effects** | exp *Teratogenesis/ OR exp *congenital disorder/ OR exp *cardiovascular malformation/ OR exp *eye malformation/ OR exp *microtia/ OR exp *musculoskeletal system malformation/ OR exp *craniofacial malformation/ OR exp *central nervous system malformation/ OR exp *respiratory tract malformation/ OR urogenital tract malformation OR exp *fetus disease/ OR exp *digestive system malformation/ OR exp *limb deformity/ OR exp *polydactyly/ OR exp *syndactyly/ OR exp *Communication Disorder/ OR exp *autism/ OR exp *intellectual impairment/ OR exp *learning disorder/ OR exp *low birth weight/ OR exp *small for date infant/ OR exp *psychomotor disorder/ OR exp *developmental disorder/ OR exp *developmental language disorder/ OR exp *fertility/ OR exp *embryo development/ OR exp *prenatal development/ OR exp *Prenatal Injury/ OR exp *Fetus Development/ OR (Teratogenesis OR teratogen OR teratogens OR Congenital Abnormalities OR Congenital Abnormality OR Deformities OR Deformity OR Congenital Defects OR Congenital Defect OR Birth Defects OR Birth Defect OR congenital anomalies OR congenital anomaly OR Cardiovascular Abnormalities OR Cardiovascular Abnormalities OR Eye Abnormalities OR Eye Abnormalities OR Congenital Microtia OR Congenital Microtias OR Anotia OR Anotias OR Microtia OR Microtias OR Musculoskeletal Abnormalities OR Musculoskeletal Abnormality OR Craniofacial Abnormalities OR Craniofacial Abnormality OR Nervous System Malformations OR Nervous System Malformation OR Nervous System Abnormalities OR Nervous System Abnormality OR Nervous System Anomalies OR Nervous System Anomaly OR Nervous System Congenital Abnormalities OR Nervous System Congenital Abnormality OR Respiratory System Abnormalities OR Respiratory System Abnormalities OR Urogenital Abnormalities OR Urogenital Abnormality OR Genitourinary Abnormalities OR Genitourinary Abnormality OR Congenital Disorders OR Congenital Disorder OR Neonatal Abnormalities OR Neonatal Abnormality OR Fetal Diseases OR Fetal Disease OR Embryopathy OR Embryopathies OR Teratogenesis OR Digestive System Abnormalities OR Digestive System Abnormality OR Drug Induced Abnormalities OR Drug-Induced Abnormalities OR Limb Deformities, Congenital OR Congenital Limb Deformities OR Congenital Limb Deformity OR Polydactyly OR Polydactylia OR Polydactylias OR Syndactyly OR Syndactylia OR Syndactylias OR Autism Spectrum Disorder OR Autism Spectrum Disorders OR Communication Disorders OR Communication Disorder OR Communicative Disorders OR Communicative Disorder OR Developmental Communication Disorder OR Developmental Communication Disorders OR Communication Disabilities OR Communication Disability OR Intellectual Disability OR Intellectual Disabilities OR Intellectual Development Disorder OR Intellectual Development Disorders OR Mental Retardation OR Psychosocial Mental Retardation OR Psychosocial Mental Retardations OR Mental Deficiencies OR Mental Deficiency OR Learning Disabilities OR Learning Disability OR Low-Birth-Weight Infant OR Low Birth Weight Infant OR Low Birth Weight Infants OR Low-Birth-Weight Infants OR Low Birth Weight OR Low Birth Weights OR Infant, Small for Gestational Age OR Motor Skills Disorders OR Motor Skills Disorder OR Developmental Coordination Disorders OR Developmental Coordination Disorder OR Attention Deficit Disorder with Hyperactivity OR Attention Deficit Disorders with Hyperactivity OR Attention Deficit Hyperactivity Disorders OR Attention Deficit-Hyperactivity Disorder OR Attention Deficit-Hyperactivity Disorders OR Hyperkinetic Syndrome OR ADHD OR Attention Deficit Hyperactivity Disorder OR Attention Deficit Disorder OR Attention Deficit Disorders OR Minimal Brain Dysfunction OR Developmental Disabilities OR Developmental Disability OR Child Development Disorder OR Child Development Disorders OR Developmental Delay Disorders OR Developmental Delay Disorder OR Child Development Deviations OR Child Development Deviation OR Language Development Disorders OR Language Development Disorder OR Developmental Language Disorders OR Developmental Language Disorder OR Speech Delay OR Speech Delays OR Semantic-Pragmatic Disorder OR Semantic Pragmatic Disorder OR Semantic-Pragmatic Disorders OR Central Auditory Processing Disorder OR Language Delay OR Language Delays OR Teratogenicity OR Post-natal development OR Fertility OR Fecundity OR Mutagenity OR Reproductive toxicity OR Developmental toxicity OR Embryonic Development OR Embryonic Developments OR Embryogenesis OR Embryo Development OR Embryonic Programming OR Embryonic Programming OR Postimplantation Embryo Development OR Post-implantation Embryo Development OR Post implantation Embryo Development OR Prenatal Injuries OR Prenatal Injury OR Prenatal Exposure Delayed Effects OR perinatal toxicity OR postnatal toxicity OR Fetal Development OR Fetal Growth OR embryo toxicity OR embryotoxicity OR fetal toxicity OR fetaltoxicity OR embryofetal toxicity OR developmental toxicity OR development toxicity).tw,kw,dy. |
| **Genetic Predisposition** | exp genetic predisposition/ OR exp genetic variation/ OR exp genetic variability/ OR exp genetic polymorphism/ OR (Genetic Predisposition to Disease OR Genetic Susceptibility OR Genetic Susceptibilities OR Genetic Predisposition OR Genetic Predispositions OR polymorphism OR polymorphisms OR Genetic Variation OR Genetic Variations OR Genetic Diversities OR Genetic Diversity OR Genetic Polymorphisms OR Genetic Polymorphism).tw,kw,dy. |

| **Appendix 4.** Names and functions of genes associated with the teratogenicity of the investigated drugs. | | |
| --- | --- | --- |
| **Gene** | **Name** | **Function** |
| *ABCB1* | ATP Binding Cassette Subfamily B Member 1 | Translocates drugs across the membrane |
| *BHMT* | Betaine-Homocysteine S-Methyltransferase | Homocysteine metabolism |
| *COMT* | Catechol-O-Methyltransferase | Catalyzes O-methylation |
| *CRBN* | Cereblon | Substrate recognition component of the DCX (DDB1-CUL4-X-box) E3 protein ligase complex; primary target of thalidomide |
| *CRHR1* | Corticotropin Releasing Hormone Receptor 1 | Activation of signal transduction pathways |
| *CYP2C19* | Cytochrome P450 Family 2 Subfamily C Member 19 | Drug metabolism |
| *CYP2D6* | Cytochrome P450 Family 2 Subfamily D Member 6 | Drug metabolism |
| *CYP3A4* | Cytochrome P450 Family 3 Subfamily A Member 4 | Drug metabolism |
| *CYP3A5* | Cytochrome P450 Family 3 Subfamily A Member 5 | Drug metabolism |
| *CYP3A7* | Cytochrome P450 Family 3 Subfamily A Member 7 | Drug metabolism |
| *EPHX1* | Epoxide Hydrolase 1 | Biotransformation enzyme |
| *GNMT* | Glycine N-Methyltransferase | Catalyzes the methylation of glycine |
| *GSTM1* | Glutathione S-Transferase Mu 1 | Catalyzes the conjugation of reduced glutathione to a broad range of hydrophobic electrophiles |
| *GSTP1* | Glutathione S-Transferase Pi 1 | Catalyzes the conjugation of reduced glutathione to a broad range of hydrophobic electrophiles |
| *GSTT1* | Glutathione S-Transferase Theta 1 | Catalyzes the conjugation of reduced glutathione to a broad range of hydrophobic electrophiles |
| *IPO13* | Importin 13 | Nuclear transport receptor |
| *MAO-A* | Monoamine Oxidase B | Oxidation of monoamines, such as dopamine, serotonin, and adrenalin |
| *MGMT* | O-6-Methylguanine-DNA Methyltransferase | DNA repair protein |
| *MGST1* | Microsomal Glutathione S-Transferase 1 | Conjugation of reduced glutathione |
| *MTHFR* | Methylenetetrahydrofolate Reductase | Catalyzes the conversion of 5,10-methylenetetrahydrofolate to 5-methyltetrahydrofolate |
| *MTHFS* | Methenyltetrahydrofolate Synthetase | Contributes to tetrahydrofolate metabolism |
| *MTR* | 5-Methyltetrahydrofolate-Homocysteine Methyltransferase | Catalyzes the final step in methionine biosynthesis |
| *NOS3* | Nitric Oxide Synthase 3 | Produces nitric oxide (NO), which acts as a mediator of the neurotransmission and angiogenesis |
| *NR3C1* | Nuclear Receptor Subfamily 3 Group C Member 1 | Glucocorticoid receptor |
| *NR3C2* | Nuclear Receptor Subfamily 3 Group C Member 2 | Mineralocorticoid receptor |
| *NR3C3* | Nuclear Receptor Subfamily 3 Group C Member 3 | Progesterone receptor |
| *NRF2* | Nuclear Factor, Erythroid 2 Like 2 | Transcription factor playing a key role in the response to oxidative stress |
| *OLR1* | Oxidized Low Density Lipoprotein Receptor 1 | Low density lipoprotein receptor |
| *SHMT1* | Serine Hydroxymethyltransferase 1 | Interconversion of serine and glycine |
| *SLC6A4* | Solute Carrier Family 6 Member 4 | Serotonin transporter |
| *SRD5A2* | Steroid 5 Alpha-Reductase 2 | Converts testosterone into 5-alpha-dihydrotestosterone and progesterone or corticosterone into their corresponding 5-alpha-3-oxosteroids |
| *TRDMT1* | TRNA Aspartic Acid Methyltransferase 1 | Methylation of aspartic acid transfer RNA (at the cytosine-38 residue) in the anticodon loop |

References: UNIPROT database (<https://www.uniprot.org/>) and GeneCards database (<https://www.genecards.org/>).
